# How Much Do We Know about Drug Resistance Due to PrEP Use? Analysis of Experts’ Opinion and Its Influence on the Projected Public Health Impact

**DOI:** 10.1371/journal.pone.0158620

**Published:** 2016-07-08

**Authors:** Dobromir T. Dimitrov, Marie-Claude Boily, Timothy B. Hallett, Jan Albert, Charles Boucher, John W. Mellors, Deenan Pillay, David A. M. C. van de Vijver

**Affiliations:** 1 Vaccine and Infectious Disease Division, Fred Hutchinson Cancer Research Center, Seattle, Washington, United States of America; 2 Department of Applied Mathematics, University of Washington, Seattle, Washington, United States of America; 3 Department of Infectious Disease Epidemiology, Imperial College London, London, United Kingdom; 4 Department of Microbiology, Tumor and Cell Biology, Karolinska Institutet, Stockholm, Sweden; 5 Department of Clinical Microbiology, Karolinska University Hospital, Stockholm, Sweden; 6 Department of Virology, Erasmus Medical Centre, University Medical Centre Rotterdam, Rotterdam, Netherlands; 7 Division of Infectious Diseases, School of Medicine, University of Pittsburgh, Pittsburgh, Pennsylvania, United States of America; 8 Research Department of Infection, University College Medical School, London, United Kingdom; University of Pittsburgh Center for Vaccine Research, UNITED STATES

## Abstract

**Background:**

Randomized controlled trials reported that pre-exposure prophylaxis (PrEP) with tenofovir and emtricitabine rarely selects for drug resistance. However, drug resistance due to PrEP is not completely understood. In daily practice, PrEP will not be used under the well-controlled conditions available in the trials, suggesting that widespread use of PrEP can result in increased drug resistance.

**Methods:**

We surveyed expert virologists with questions about biological assumptions regarding drug resistance due to PrEP use. The influence of these assumptions on the prevalence of drug resistance and the fraction of HIV transmitted resistance was studied with a mathematical model. For comparability, 50% PrEP-coverage of and 90% per-act efficacy of PrEP in preventing HIV acquisition are assumed in all simulations.

**Results:**

Virologists disagreed on the following: the time until resistance emergence (range: 20–180 days) in infected PrEP users with breakthrough HIV infections; the efficacy of PrEP against drug-resistant HIV (25%-90%); and the likelihood of resistance acquisition upon transmission (10%-75%). These differences translate into projections of 0.6%- 1% and 3.5%—6% infected individuals with detectable resistance 10 years after introducing PrEP, assuming 100% and 50% adherence, respectively. The rate of resistance emergence following breakthrough HIV infection and the rate of resistance reversion after PrEP use is discontinued, were the factors identified as most influential on the expected resistance associated with PrEP. Importantly, 17–23% infected individuals could virologically fail treatment as a result of past PrEP use or transmitted resistance to PrEP with moderate adherence.

**Conclusions:**

There is no broad consensus on quantification of key biological processes that underpin the emergence of PrEP-associated drug resistance. Despite this, the contribution of PrEP use to the prevalence of the detectable drug resistance is expected to be small. However, individuals who become infected despite the use of PrEP should be closely monitored due to higher risk of virological failure when initiating antiretroviral treatment in the future.

## Introduction

Pre-exposure prophylaxis (PrEP) has garnered significant attention from the HIV research community after efficacy was demonstrated in different populations. [1–6] There is a concern that widespread use of PrEP can result in drug resistance, which in turn can limit future treatment options. [7] HIV has a high mutation rate which allows the virus to quickly adapt to treatment by selecting drug resistance associated mutations. Only a single mutation in the viral genome is required for resistance to tenofovir (K65R), and another single mutation is required for resistance to emtricitabine (M184V). [8] Resistance is avoided by prescribing a drug regimen in which the virus requires a sufficiently high number of mutations to overcome drug selective pressure. Such a regimen, which has a high genetic barrier, [9] generally consists of a combination of three different drugs from two different classes of antiretroviral drugs. PrEP, which consists of at most two drugs, may have a too-low genetic barrier. Use of PrEP may, therefore, result in rapid emergence of resistance.

In clinical trials to date, HIV drug resistance was found in <0.5% of patients that were randomized to PrEP and who became infected with HIV. Drug resistance developed predominantly among participants with unrecognized acute HIV infection who were randomized to start PrEP. [10–12] Though this is somewhat reassuring, uncertainty remains regarding the resistance risk once PrEP is implemented widely; patients prescribed PrEP may not have the close monitoring and frequent HIV-testing mandated in clinical trials. As a consequence, a new HIV-infection might not be identified in a timely manner and patients who continue using PrEP may select for drug resistance. [7] Experience with early options for antiretroviral treatment (ART) suggests that if HIV-positive individuals are continuously exposed to only one or two active drugs, emergence of drug resistance should be expected. [13,14]

HIV drug resistance due to PrEP is complex and influenced by different processes (Table 1). First, acquired drug resistance (ADR) can emerge in individuals who continue using PrEP after they become infected with HIV [10–12]. Second, transmitted drug resistance (TDR) occurs when an individual becomes infected with a drug resistant virus. [15] Third, in the absence of antiretroviral drugs, a resistant virus can revert to a drug susceptible wild-type virus. [16] Importantly, individuals in whom reversion of resistance has occurred have a higher risk of virological failure as compared to individuals that have been infected with a wild-type virus [17]. Resistance to drugs used as PrEP (i.e., tenofovir and emtricitabine) has been investigated only in combination with a third antiretroviral drug. [18–20] As a consequence, the processes involved in drug resistance due to PrEP are difficult to parameterize using available evidence from the literature.

**Table 1:** Key mechanisms surveyed to inform modeling assumptions about PrEP-associated resistance.

Resistance mechanism surveyed	Description, background and model parameters informed	Estimates based on our survey*
Resistance emergence in infected individuals still using PrEP	The use of ARV as PrEP by infected individuals leads to emergence of drug resistance (acquired drug resistance, ADR) because PrEP is not designed for treatment and is unlikely to exert complete HIV suppression [24,26,30,34,35,56]. The rate of resistance emergence may depend on the consistency of PrEP use (adherence) since it controls the ARV drug concentrations and from there the pressure on the HIV virus. [47]	
	Rate of resistance development with perfect adherence	2–18
	Rate of resistance development with intermediate (low)** adherence	2–18 (0.66–24)
Reversion of resistance	HIV containing resistance associated mutations against PrEP can revert to wild-type when PrEP is discontinued or when an ARV-naïve host is infected with resistant HIV [24,26,34,35]. The rate of reversion may be substantially slower in ARV-naïve individuals with TDR compared to former PrEP users who developed ADR on PrEP [57–59]. Future treatment is affected because resistant viruses can remain present as minority variants, which can increase the risk of virological failure.	
	Rate of resistance reversion in former PrEP users who acquired drug resistance when on PrEP	1.33–8
	Rate of resistance reversion for PrEP naïve to whom the resistant HIV has been transmitted	0.2–1.5
Reduced fitness of the drug-resistant HIV	Resistance carriers may be less infectious than those infected with wild-type HIV as a result of the reduced replication capacity [24,26,30,34,35,56] of the resistant HIV, which in turn can reduce the viral load. It may result in lower viral concentration that affects the likelihood of transmission. [47]	
	Relative infectiousness of individuals with ADR	0.4–1
	Relative infectiousness of individuals with TDR	0.7–1
Transmission of resistance	When infections from contacts with resistance carriers occur, either resistant or wild-type HIV may be transmitted. Individuals who carry transmitted resistance may be more likely to transmit drug-resistance compared to those who have developed resistance on PrEP. [24,26,57]	
	Probability to transmit ADR by PrEP status of both partners [inf. -> susc.]:	
	on PrEP→off PrEP	0.09–0.75
	on PrEP →on PrEP	0.17–0.91
	off PrEP →off PrEP	0.09–0.5
	off PrEP →on PrEP	0.09–0.5
	Probability to transmit TDR	0.2–0.6
PrEP efficacy against drug-resistant HIV	PrEP may provide reduced protection against PrEP-generated resistance since those HIV strains have managed to escape the ARV pressure of PrEP in infected users [24,26,34,35]. Reduced effectiveness of PrEP has been suggested by studies in macaques challenged with resistant SIV. [60]	
	PrEP efficacy when exposed to ADR relative to wild type HIV	0.25–0.9

*Ranges represent the variation in point estimates across participants. Detailed description of all responses is presented in the Table 2. All rates are on annual basis.

**Intermediate and low adherence correspond to half (50%) and one weekly (14%) PrEP doses taken, respectively.

Mathematical models have helped to evaluate the potential benefits of PrEP interventions in different populations and some of them have been used to predict the level of drug resistance generated by PrEP use. [21–43] An analytic review of published modeling studies on the impact of PrEP highlighted that modeling assumptions regarding resistance had a strong influence on the predicted long-term effectiveness of PrEP. [44] Examined under identical epidemic conditions, those assumptions resulted in PrEP-associated resistance prevalence between 2% and 44% after 10 years of PrEP use. A comparative study of three independent mathematical models on the impact of PrEP on HIV transmission and drug resistance in sub-Saharan African countries concluded that PrEP will increase the relative prevalence of drug resistance amongst infected individuals by at most 7% over 20 years if PrEP users are frequently tested for HIV (every 3–6 months). [45] It is not clear whether disparate modeling results reflect underlying different views on existing empirical data. Difference in the existing levels of resistance when PrEP is rolled out has been suggested as a possible explanation of conflicting modeling predictions. [46] However, with many aspects of resistance unknown, [45,47] the impact of any individual assumption is difficult to isolate.

**Fig 1:**
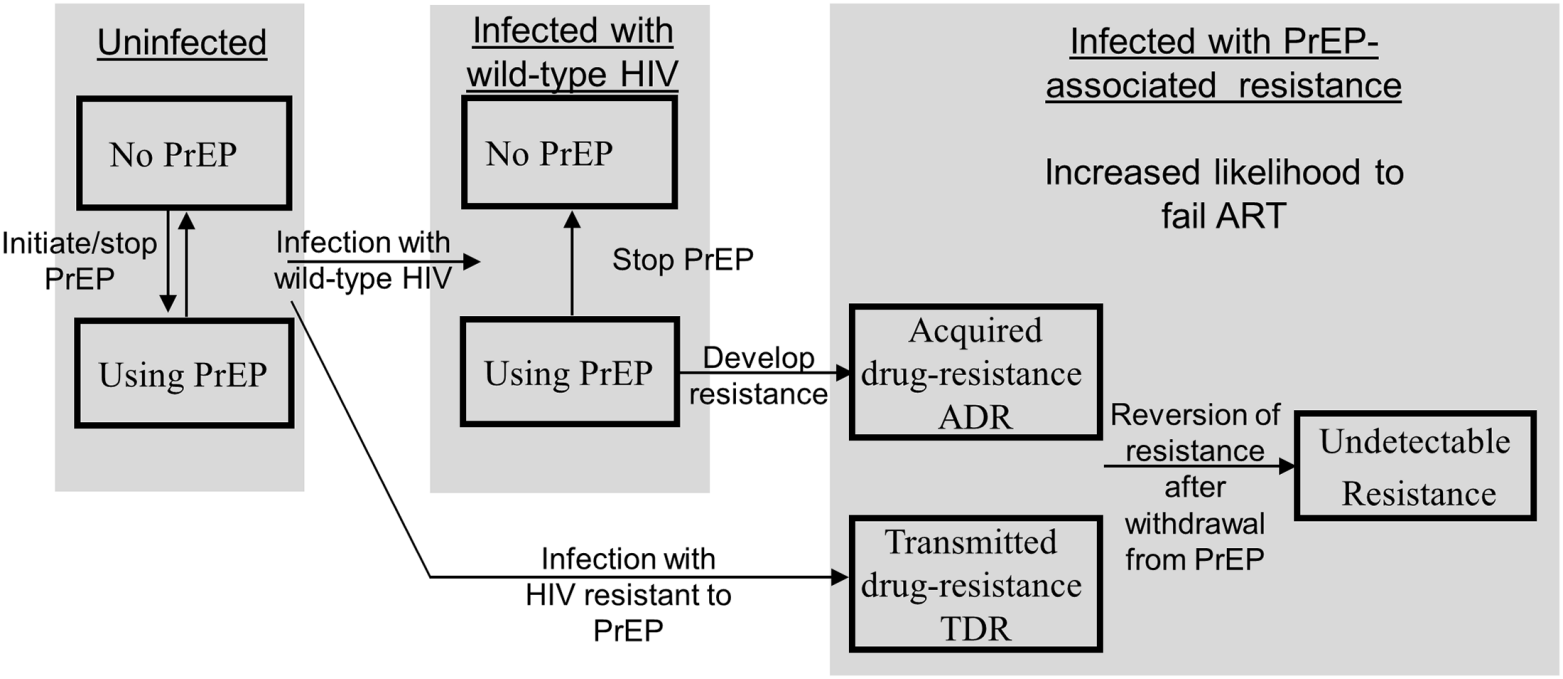
Diagram of emergence and transmission of PrEP-associated drug-resistance. The diagram shows how resistance can occur in the presence of PrEP. The boxes represent different states in which individuals can be divided. These states include the use of PrEP by uninfected and infected individuals, as well as infected and HIV-infected individuals who do not use PrEP and HIV-infected individuals who carry PrEP-associated drug resistance. The processes contributing to PrEP-associated resistance include 1) transmission of resistance in which individuals become exposed to and infected with drug-resistant HIV (transmitted drug resistance, TDR), and 2) resistance that occur when individuals continue the use of PrEP after they become infected (acquired drug resistance, ADR). PrEP-associated resistance may drop below detectable level in individuals not exposed to PrEP for long period of time. They may lose the ability to transmit resistance but remain at elevated risk to fail ART when initiated.

We conducted a survey of expert virologists to evaluate current opinions on the mechanisms of generating PrEP-associated drug resistance (Fig 1) and used it to inform the resistance assumptions integrated in mathematical models. Our goal was to determine whether major points of disagreement between virologists exist and how these differences propagate into modeling projections. Key areas where empirical data is critically needed are outlined based on the results of our analysis.

## Methods

### Virologist survey

Eleven experts from the US and Europe who have studied drug resistance mechanisms at the clinical, virological, biochemical and structural levels were identified through recommendations from the HIV Prevention Trials Network, the Microbicide Trials Network and the HIV Modeling Consortium. They were invited to answer a questionnaire on emergence, persistence, and transmission of HIV drug resistance due to PrEP use. The survey was created to inform key mechanistic assumptions used in mathematical models (see Table 1). One expert (DV) reviewed the questionnaire to ensure that no critical information was omitted and to advise the proper formulation of each study item. Experts were invited to participate by e-mail and informed that the questionnaire was for the purpose of research use, that the survey results were going to be analyzed and written for publication. They were asked to provide quantitative estimates (specific values and uncertainty ranges) on each assumption based on their best knowledge and were given an option to explain the rationale for their answers. All responders provided opinion independently with no structured communication methods applied. The actual survey template is included in the S2 Text.

Modeling studies investigated drug resistance when PrEP is integrated with an active ART program and concluded that the vast majority of the resistance cases can be attributed to ART. [34,45] Our survey outlines the complexity of the processes of resistance emergence associated with PrEP (see Fig 1); therefore, the analysis focuses on the introduction and use of PrEP only without modeling its interactions with ART.

### Mathematical model

In order to assess the influence of resistance assumptions on the outcomes of the PrEP intervention, we developed a mathematical model (see model diagram, Fig A in S1 Text) to simulate generalized heterosexual HIV epidemics in Sub-Saharan Africa. The individuals in the simulated population are aggregated in compartments by gender (men and women), by PrEP status (users and nonusers), and by HIV status (uninfected, infected with wild-type HIV, infected with ADR, and infected with TDR). Susceptible men and women who become sexually active join the community at constant rates, corresponding to 2% population growth in uninfected populations. The rates at which individuals acquire HIV infection depend on the annual number of partners per susceptible person, the number of sex acts per partnership, the fraction of sex acts protected by condoms, and the HIV acquisition risk per vaginal act for men and women, which may depend on resistance status of the infected partner at the time of exposure (i.e., viremia with wild type vs. drug-resistant HIV). A limited access to PrEP by HIV-positive individuals and periodic HIV screening, which interrupts PrEP use by infected individuals after an average of 1 year, is assumed. We have employed a frequently used modeling assumption that PrEP efficacy, i.e, the reduction in susceptibility to HIV, is proportional to the adherence. [26,37,38,48] Scenarios exploring different frequency of HIV testing and residual PrEP protection on days when it is not taken (suggested in [43,49]), are investigated in the S1 Text.

In order to calibrate the model, we fit its outcomes to the HIV prevalence and HIV incidence reported in South Africa (find calibration procedure in the S1 Text). Demographic, behavioral and epidemic parameters are informed by statistical data from South Africa and other published sources (see Table A in S1 Text). Parameters related to the PrEP-associated resistance are informed by responses to our survey (Tables 1 and 2). The model is used to simulate HIV epidemics in absence of PrEP to provide a baseline for the evaluation of the PrEP impact. Simulations assuming that that PrEP use does not lead to drug resistance serve as a baseline when the impact of the resistance assumptions is evaluated. Finally, model simulations parameterized with the survey data are used to compare the resistance assumptions suggested by virologists.

**Table 2:** Resistance parameters sets based on the point estimates (ranges) provided by the virologists.

Parameters	V1	V2	V3	V4	V5	Aggregated set*
**Rate of resistance development with perfect adherence**	12 (4–52)	12	18 (12–24)	4 (2–12)	2 (1–4)	11.76 (1.82–30)
Rate of resistance development with intermediate (low)** adherence	0.66 (4)	No answer	1.2 (18)	24 (8)	0.66 (2)	Linear dependence on adherence is explored in the sensitivity analysis
**Rate of resistance reversion for former PrEP users who acquired drug resistance when on PrEP**	4	8	4	1.33–2	2	4 (1.33–8)
**Rate of resistance reversion for PrEP naïve to whom the resistant HIV has been transmitted**	0.5	1	1.5 (1–2)	0.2 (0.17–0.25)	0.2 (0.17–0.25)	0.5 (0.17–2)
**Probability to transmit ADR by PrEP status [inf. -> susc.]**:						
**on PrEP→off PrEP**	0.09 (0.01–0.17)	0.38 (0.23–0.5)	0.41 (0.38–0.44)	0.5 (0.33–0.75)	0.75 (0.67–0.89)	0.41 (0.07–0.8)
**on PrEP→on PrEP**	0.5 (0.33–0.67)	No answer	0.17 (0.13–0.2)	0.5 (0.33–0.67)	0.91 (0.83–0.94)	0.5 (0.15–0.91
**off PrEP →off PrEP**	0.09 (0.01–0.11)	0.23 (0.2–0.29	0.09 (0.01–0.11)	0.5 (0.33–0.67)	0.09 (0.01–0.17)	0.1 (0.04–0.55)
**off PrEP →on PrEP**	0.09 (0.01–0.17)	0.38 (0.33–0.41)	0.09 (0.01–0.11)	0.2 (0.09–0.31)	0.5 (0.33–0.67)	0.2 (0.04–0.55)
**Probability to transmit TDR**	0.5 (0.4–0.6)	0.5 (0.45–0.55)	0.5 (0.4–0.6)	0.6 (0.5–0.7)	0.2 (0.05–0.35)	0.5 (0.16–0.63)
**PrEP efficacy when exposed to ADR relative to wild type HIV**	0.25 (0–0.5)	0.6 (0.5–0.7)	0.9 (0.8–1)	0.25 (0–0.5)	0.25 (0.2–0.4)	0.34 (0.12–0.93)
**Relative infectiousness of individuals with ADR**	1 (0.8–1.2)	0.4 (0.3–0.5)	0.9 (0.8–1)	1	1	0.95 (0.37–1.07)
**Relative infectiousness of individuals with TDR**	1 (0.9–1.1)	0.7 (0.5–0.9)	0.95 (0.9–1)	1	1	0.97 (0.64–1.06)

*The ranges represent the 90% confidence level of distributions pooled from the experts’ estimates (find pooling procedure in the S1 Text).

**Intermediate and low adherence correspond to half (50%) and one weekly (14%) PrEP doses taken, respectively.

Two widely used metrics are evaluated to project the impact of PrEP use on the spread of resistance over 10 years: the prevalence of drug-resistance among HIV-positive individuals and the cumulative fraction of infections in which drug-resistant HIV is transmitted. Additionally, we estimated the proportion of infected individuals with elevated risk to fail ART due to PrEP-associated resistance. In this respect, we have included a question about reversion of drug resistance in the survey. Reversion occurs when HIV mutates to a drug-susceptible wild-type virus in the absence of drug selective pressure. Patients, in whom reversion occurred, will not transmit a drug resistant virus. Nonetheless, such patients have an increased risk for virological failure as a minority resistant variant can rapidly reemerge after a future start of a regimen that includes an antiretroviral drug to which resistance exists. [50]

We study the uncertainty in the intervention metrics generated by the individual resistance factors in multivariate sensitivity analyses. All the parameters are varied across ranges aggregated from the survey responses but without taking each set of parameter estimates together. A complete description of the model is included in S1 Text.

## Results

### Survey results: How virologists describe drug-resistance due to PrEP?

Six experts who received our survey responded with quantitative estimates (two experts submitted a joint opinion) and have been included in this analysis. All responders, whose names and affiliations are included in the S1 Text, agreed their estimates to be used in the analysis. The responses were anonymized prior their inclusion in the analysis and projections are presented in the figures in no particular order. The values and ranges of the resistance parameters informed by the survey responses are presented in Tables 1 and 2. Projections associated with the M184V mutation have been used in the analysis, as this mutation is the most common mutation found in individuals that become infected despite the use of PrEP [10–12]. The aggregated parameter set consists of ranges representing the 90% confidence level of distributions pooled from values and ranges suggested by the experts (find pooling procedure in the S1 Text). It has been used in the sensitivity analyses to study the influence of single resistance parameters or groups of related parameters on different intervention outcomes.

**Fig 2:**
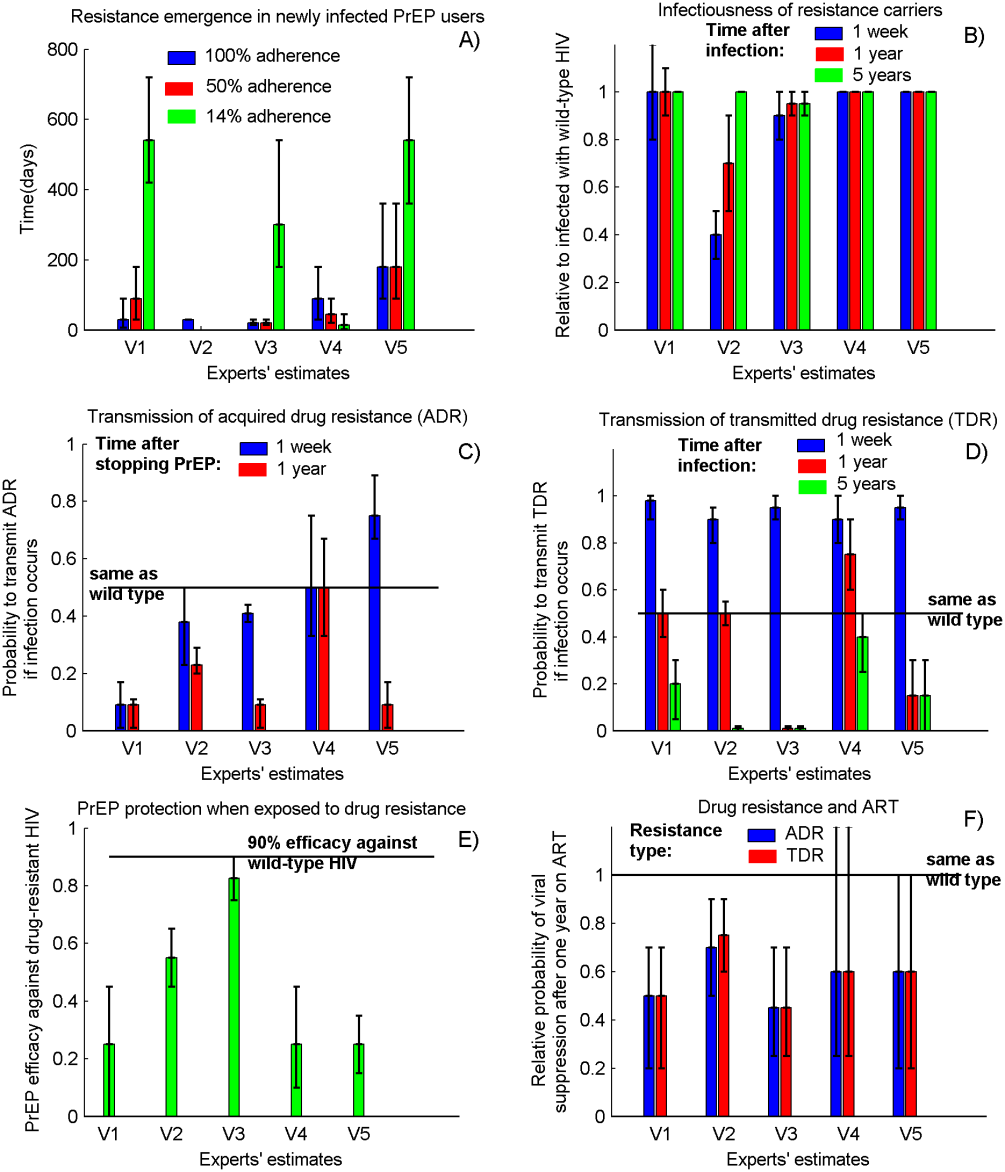
Emergence and transmission of PrEP-associated resistance predicted by the virologists. A) rate of resistance emergence in infected PrEP users; B) relative infectiousness of the resistance carriers compared to infected with wild-type HIV; C) relative chance to transmit drug-resistant over wild-type HIV if resistance is acquired on PrEP (ADR); D) relative chance to transmit drug-resistant over wild-type HIV if resistance is acquired through transmission (TDR); E) PrEP protection against drug-resistant HIV and F) relative chance for viral suppression of the resistance carriers when ART is initiated. The bars (whiskers) represent the mean estimate (range) predicted by each respondent. Complete description of the survey results is provided in the Table 2.

Comparison of the key responses to our survey are presented in Fig 2 while virologists’ opinions on specific assumptions of PrEP-generated resistance are summarized below.

#### Rate of resistance emergence in newly infected PrEP users

Virologists believed that resistance emerges between 20 and 180 days of consistent PrEP use (perfect adherence) by infected individuals (Fig 2A, blue bars). They agreed that adherence to PrEP may affect the rate of resistance emergence in HIV-infected PrEP users but did not all agree on the threshold below which the drug level is insufficient to apply a meaningful selective pressure for development of resistance. Three experts suggested that low adherence (taking PrEP once a week) will apply weak selective pressure, whereas one virologist believed that it will be sufficient to elicit rapid development of resistance. Both high and low ADR emergence rates were therefore deemed plausible for low adherence to PrEP, resulting in a wide range (15–540 days) of estimated time to resistance (Fig 2A, green bars).

#### Reversion of drug resistance

Experts agreed that when individuals who carry resistant viruses are unexposed to PrEP for long periods of time, the proportion of drug-resistant viruses in their blood will drop below the limit of detection (Table 2). They suggest that the process of reversion takes longer for TDR (estimates range 6–60 months) than for ADR (1.5–9 months) to become undetectable in plasma.

#### Infectiousness of infected with a drug resistant HIV

Three of the five respondents indicated that individuals infected with drug-resistant HIV through transmission are as likely to infect others as those infected with wild-type (Fig 2B). The two remaining experts predict that the infectiousness of TDR carriers may be reduced by 10–60% over the first weeks and by 5–30% over the first year after the HIV acquisition. Based on the differences in the rate of TDR and ADR reversion, the average infectiousness of the TDR carriers is assumed similar to the infectiousness of TDR carriers one year after the acquisition of HIV while the average infectiousness of the ADR carriers is assumed similar to the infectiousness of TDR carriers shortly after the acquisition of HIV.

#### Probability to transmit ADR

Three respondents believed that shortly after stopping PrEP, viruses with acquired drug resistance are less likely to be transmitted to HIV-uninfected PrEP naïve partners, compared with wild-type viruses (Fig 2C). They predicted that the probability to transmit resistance is higher shortly after stopping PrEP (9–41%) compared to a year after (9–23%). One virologist suggested that wild-type HIV is substantially less likely to be transmitted by recent PrEP users while another believed that the chance to transmit resistant HIV is the same as wild-type HIV. In our setup the likelihood of resistance transmission from active PrEP users was assumed similar to the likelihood shortly after stopping PrEP.

#### Probability to transmit TDR

All experts suggested that shortly after an infection with resistant HIV the likelihood of resistance transmission is almost certain (Fig 2D, blue bars). However, they disagreed on the likelihood resistant HIV to be transmitted one year after HIV acquisition (range 1%-75%). Given the estimated persistence of TDR (6–60 months) we used those 1-year projections to approximate the likelihood of TDR transmission.

#### Efficacy of PrEP against resistant HIV

Although their quantitative estimates varied, virologists agreed that exposure to PrEP-associated resistance will reduce the efficacy of PrEP (Fig 2E). They predicted that PrEP, which is 90% effective against wild-type HIV, may provide between 25% and 80% protection against resistant HIV.

#### Survival and disease progression when infected with drug-resistant HIV

All respondents agreed that resistance (both ADR and TDR) will not affect the progression of the HIV infection in absence of ART (see Table 2). They also agreed that the effectiveness of NRTI-based ART will be compromised for those who show drug-resistance to PrEP (Fig 2F). They estimated that resistance carriers may be 25–55% less likely to achieve viral suppression after 1 year on ART, which shares active components with PrEP.

A complete description of all projections provided by the virologists is included in Table 2.

### Public health impact of PrEP based on the responses to the survey

**Fig 3:**
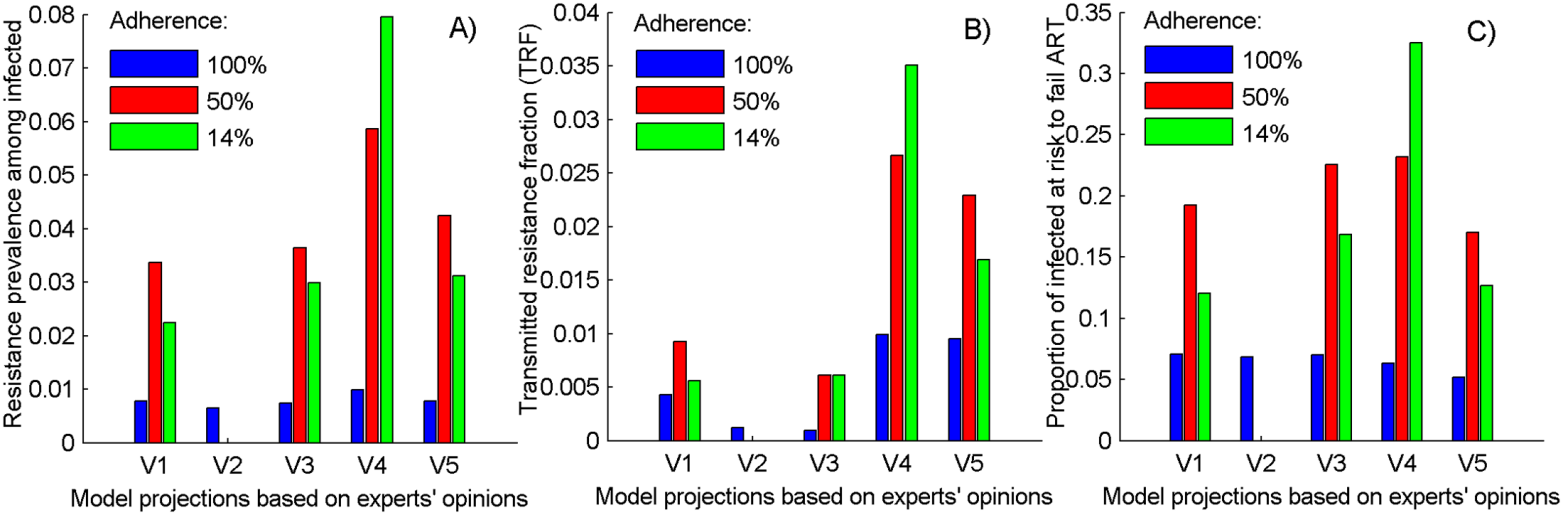
Projections on the expected drug-resistance after 10 years of PrEP use based on the virologists’ opinion. A) resistance prevalence due to PrEP; B) cumulative fraction of infections in which resistance is transmitted (TRF) and C) the fraction of infected individuals with elevated risk to fail ART. The model is parameterized with the responses to the survey, assuming different levels of adherence to PrEP. The bars represent the mean metrics estimates based on 1,000 epidemics simulated. Intervention parameters are fixed on their baseline values from Table A, part 3 in S1 Text.

Fig 3 presents model projections when parameterized with the resistance parameters suggested by each respondent to our survey. It shows that 10 years after PrEP introduction, the prevalence of resistance (combined ADR and TDR) among the infected individuals varies across virologists and adherence assumed (range 1%—8%). Larger prevalence of resistance should be expected for intermediate and low adherence (Fig 3A, red and green columns). This may be important given that detection of study drugs in the samples of completed clinical trials suggests that the majority of participants use PrEP inconsistently. [1,51] The elevated projections associated with the simulation set V4 are results of the rapid emergence of ADR combined with the lowest reversion rates and the worst PrEP protection against resistant HIV assumed (see Fig 2A and 2E).

Little variation in the fraction of infections in which resistance is transmitted over a 10-year period (transmitted resistance fraction, TRF) is projected if PrEP is consistently used with estimates below 1% (Fig 3B, blue columns). Two of the simulation sets (V4 and V5) suggest that TRF may be sensitive to the level of adherence. A combination of three key assumptions explains the larger TRF associated with sets V4 and V5: high probabilities of resistance transmission by recent PrEP users; equal infectiousness when infected with resistant and wild-type HIV; and low PrEP efficacy against resistant HIV (see Fig 2B, 2C and 2E).

**Fig 4:**
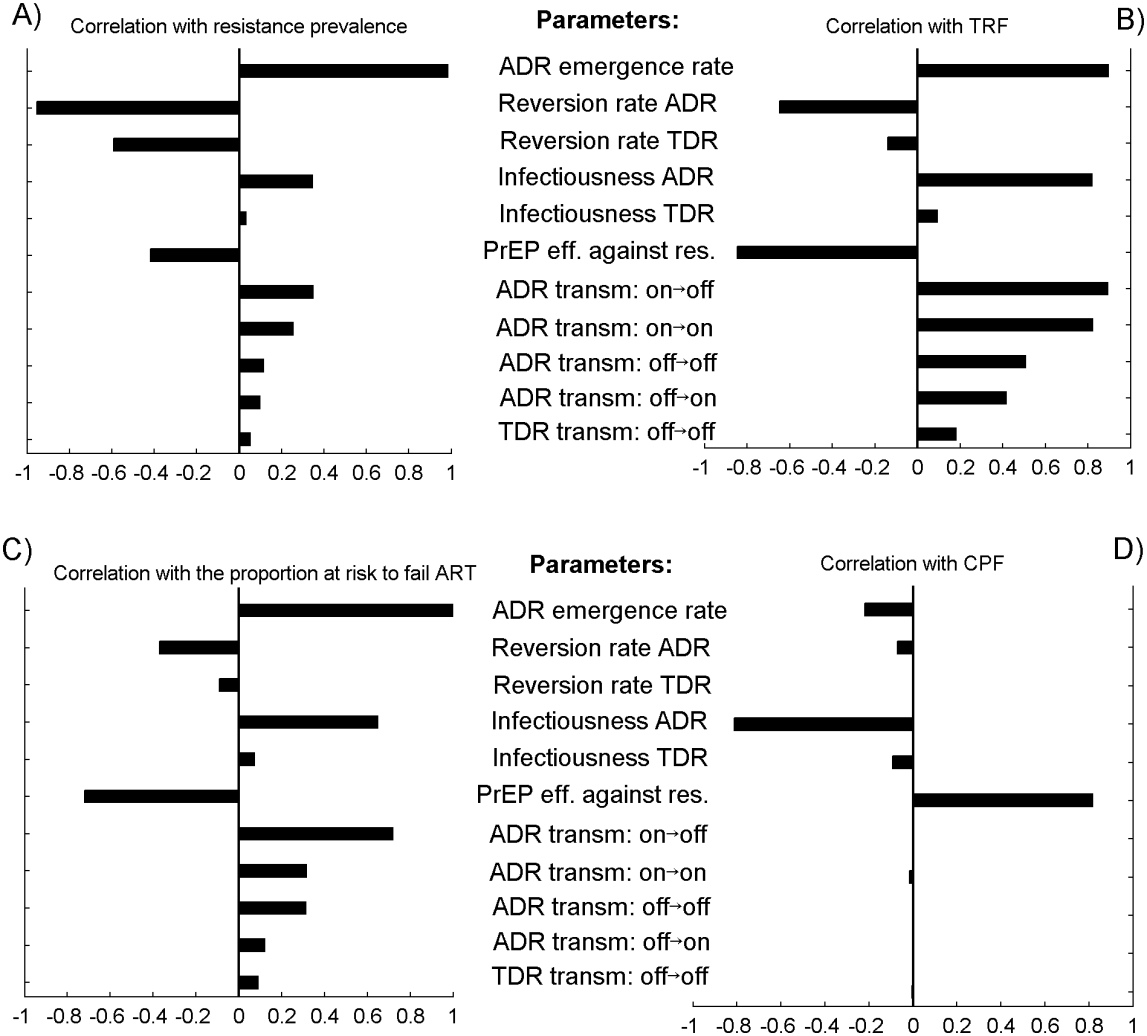
Results from multivariate sensitivity analysis. Partial rank correlation coefficients (PRCC) between resistance parameters and intervention metrics: A) resistance prevalence due to PrEP (RP), B) cumulative fraction of infections in which resistance is transmitted (TRF), C) the fraction of infected individuals with elevated risk to fail ART and D) cumulative fraction of prevented infections (10-year CPF) and. Resistance parameters are sampled from their pooled ranges based on the responses to the virologists survey (Table 2). Linear increase of the ADR emergence rate with adherence to PrEP and PrEP efficacy per act proportional to adherence are assumed. Variation in the probabilities of ADR and TDR with respect of who is using PrEP when transmission occurs is considered. For instance (ADR transm: on->off) denotes the probability that ADR is transmitted from a PrEP user to a non-user.

Our analysis suggests that assuming imperfect adherence to PrEP, a substantial fraction of individuals may be at increased risk for virological failure at the end of a 10-year period as they become infected despite the use of PrEP or by direct exposure to resistant HIV. In these individuals, resistance may be present as minority variants that cannot be measured using population sequencing and negatively impact ART treatment. We estimate that this may be a problem for 17% to 24% of all HIV-infected individuals when 50% adherence is assumed and for 13% to 33% if PrEP is taken once weekly (Fig 3C, red and green columns). This proportion could be reduced to levels below 10% with 50% adherence if more frequent HIV testing is assumed (see Fig D in S1 Text) and if PrEP is more forgiving of missing doses (see Fig E in S1 Text) as suggested by the iPrEX team for use by MSM. [49] The main factor contributing to this metric is the resistance emergence rate (see Fig 2A) as confirmed by sensitivity analysis (see Fig 4 and Fig C in S1 Text).

Despite substantial variation in virologists’ opinions (Fig 2), little differences in the reduction of HIV prevalence and the fraction of new infections averted over 10 years are expected for all adherence levels (see Fig B in S1 Text).

### Influence of resistance parameters on the intervention metrics

The results of the multivariate sensitivity analysis, summarized in Table 3, suggest that parameters that strongly influence the prevalence of PrEP-associated resistance among infected individuals after 10 years of PrEP use in the population are the rates of resistance emergence and reversion of ADR (Fig 4A). Together they explain 95% of the variation in this metric. The fraction of transmitted resistance (TRF) is primarily influenced by the parameters that determine the fitness of the resistant HIV such as the infectiousness of the resistance carriers and the transmissibility of the ADR (~60% of the variance explained). The strongest driver of uncertainty in the fraction at risk to fail ART is the ADR emergence rate (Fig 4B), which accounts for more than 98% of the variance of the metric. Detailed descriptions of the performed sensitivity analyses are included in the S1 Text.

**Table 3:** Dependence of the intervention outcomes on the resistance parameters.

Intervention outcome	Projected range after 10 years of PrEP use*	Importance of resistance assumptions	Most sensitive to (varience explained**)
Fraction of HIV infected at risk to fail ART due to PrEP-associated resistance to	12–24%	high	ADR emergence rate (99%)
Prevalence of resistance among infected individuals (RP)	2–4.5%	moderate	ADR emergence rate (55%), ADR reversion rate (40%)
Fraction of infections with transmitted resistance (TRF)	0.5–2.5%	moderate	Probability to transmit ADR over wild- type HIV (47%), Relative effectiveness against resistant HIV (19%), Relative infectiousness of ADR carriers (43%)
Cumulative fraction of infections prevented (CPF)	29–32%	low	Relative effectiveness against resistant HIV (51%), Relative infectiousness of ADR carriers (43%)

* Assuming that 90% PrEP is used by 50% of the population with 50% adherence

** See Table B in S1 Text for more details

## Discussion

Limited human data exists on PrEP-associated drug resistance while different interpretations of animal data appear to support slightly different models about the biology of the emergence and persistence of drug resistance in breakthrough infections. We have surveyed the knowledge on the topic, translated experts’ opinion into modeling assumptions and objectively compared the importance of resistance in the course of PrEP intervention under a common modeling framework. We outlined the biological factors with the strongest influence on the detectable and undetectable resistance prevalence attributed to PrEP.

We found that experts disagreed on how fast resistance develops in PrEP users unaware of being infected, how likely it is for the resistance to be transmitted, how much protection PrEP provides against resistant HIV strains and how fast the dominance of wild-type HIV is restored after PrEP use is interrupted. Experts agreed on principle as to what the mechanisms of resistance emergence are but provided a wide range of values when quantitative estimates were expected. As illustration, they all agreed that PrEP will be less efficient against PrEP-associated resistance but their estimates of the magnitute of the reduced protection varied substantially.

We have potentially amplified the impact of these uncertainties by assuming high population coverage (50%) and proportional reduction of PrEP efficacy with imperfect adherence which increases the likelihood for breakthrough infections and allows us to access the worst-case scenario of the expected amount of resistance. Regardless, we found that the differences in the modeling projections of detectable resistance prevalence based on the experts’ responses were small despite the disparity in these opinions. Our analysis suggests 1%-8% prevalence of resistance after PrEP has been used for 10 years with different levels of adherence assumed. These results are comparable with the projections from a model-comparison study in which the prevalence of drug resistance amongst infected individuals is expected to increase by at most 7% over 20 years due to PrEP use. [45]

Conversely, the model suggests substantial uncertainty in the proportion of infected individuals who may carry resistance in undetectable minority variants due to interrupted PrEP use or transmission of resistant HIV. Individuals with minority resistant variants are unlikely to transmit resistance to others. Previous studies have, however, shown that the presence of minority resistance variants can increase the risk of virological failure when ART is initiated. [52–55] Our estimates of this fraction (17–24%) under intermediate adherence to PrEP indicate that even a low level of PrEP resistance detected in the early stages of the intervention may generate a large number of ART failures due to PrEP at later stages (after 8–10 years). The extent to which minority variants that emerge as a consequence of PrEP exposure affect future treatment failure is not known. Studies investigating NNRTI resistance, however, found that 35% of patients infected with a minority drug resistant NNRTI variant experienced virological failure. [52] Our findings may not be critical if PrEP does not share active components with the available ART options or if no cross-drug resistant mutations have sufficient fitness to persist after ART is initiated. Moreover, the proportion of infected individuals at risk to fail ART may be reduced if PrEP is more forgiving on missing doses as we demonstrated in alternative scenarios. Nevertheless, the breakthrough HIV infections of PrEP users need to be monitored continuously, especially in regions where the first-line ART is TDF based.

In a supplementary analysis we demonstrated that the projected resistance prevalence (detectable or not) is substantially reduced when efforts are made to improve PrEP adherence, to prevent infected people from initiating PrEP and to periodically test PrEP users for HIV (see Fig D in S1 Text). It is encouraging that a wide-scale PrEP intervention can be implemented without facing problems with resistance even if the most pessimistic projections of our experts happen to be true. It also suggests that it is critical to identify risk subpopulations that will consistently take PrEP prior to intervention being rolled out and to integrate reliable monitoring of the HIV status of the PrEP users into the prevention program. Alternative scenarios suggest that expected PrEP-associated resistance may be reduced substantially if intermediate adherence is sufficient for protection.

Our model was structured to accommodate the biological processes related to the occurence, persistence and transmission of drug-resistance due to PrEP use. It provided a platform for objective comparison of the assumptions proposed by the virologists and for evaluation of the influence of each assumption, independently. Some of the simplifying assumptions integrated in the model may affect the PrEP impact projections. For instance, antiretroviral treatment of the infected individuals and the resistance associated with it was not modeled separately. Therefore, the projected resistance prevalence here should be considered in addition to the resistance circulating in the population as a result of ART. No heterogeneity in the adherence to PrEP and in the emergence rate of HIV mutations insensitive to PrEP has been explored. This may be an important, future research direction, which could be addressed by employing individual-based modeling approaches. [43] Our focus on heterosexual epidemics in Sub-Saharan Africa limits the usefulness of this analysis to epidemics in resource-rich settings (US and Europe) where the majority of HIV infections occur among men who have sex with men.

In conclusion, limited knowledge is available regarding the impact of PrEP on drug resistance. In this paper, we have, therefore, filled the knowledge gaps by surveying expert virologists about the potential contribution of PrEP to drug resistance. Although there was no broad consensus on the exact contribution of particular processes, it was shown that the impact of PrEP on the prevalence of resistance was fairly limited. Resistance should therefore not be a reason to withhold PrEP. Based on expert opinion, we do, however, predict that patients who become infected despite the use of PrEP may have an increased risk for virological failure. Therefore, PrEP users should be routinely tested for HIV and patients that become infected despite the use of PrEP should be closely monitored for virological failure.

## Supporting Information

S1 Text Technical Appendix. Complete model description, parameterization and calibration procedures, additional results and sensitivity analyses.
(DOCX)

S2 Text Survey Template. Template of the questionnaire on emergence, persistence, and transmission of HIV drug resistance due to PrEP use used in the study.
(PDF)
