# Supplementary material for: How Much Do We Know about Drug Resistance Due to PrEP Use? Analysis of Experts’ Opinion and Its Influence on the Projected Public Health Impact
Source: PLoS One. 2016 Jul 8;11(7):e0158620. doi: 10.1371/journal.pone.0158620 (PMC4938235; doi:10.1371/journal.pone.0158620)
Supplement: S1 Text — Complete model description, parameterization and calibration procedures, additional results and sensitivity analyses. (DOCX) [file pone.0158620.s001.docx]

**Supporting information**

**How much do we know about drug resistance due to PrEP use? Analysis of experts’ opinion and its influence on the projected public health impact**

Dobromir T. Dimitrov^1,2,*^, Marie-Claude Boily^3^, Timothy B. Hallett^3^, Jan Albert^4,5^, Charles Boucher^6^, John W. Mellors^7^, Deenan Pillay^8^, David A.M.C. van de Vijver^6^

^1^Vaccine and Infectious Disease Division, Fred Hutchinson Cancer Research Center, Seattle, Washington, USA

^2^Department of Applied Mathematics, University of Washington, Seattle, Washington, USA

^3^Department of Infectious Disease Epidemiology, Imperial College London, London, UK

^4^Department of Microbiology, Tumor and Cell Biology, Karolinska Institutet, Stockholm, Sweden

^5^Department of Clinical Microbiology, Karolinska University Hospital, Stockholm, Sweden

^6^Department of Virology, Erasmus Medical Centre, University Medical Centre Rotterdam, Rotterdam, Netherlands

^7^Division of Infectious Diseases, School of Medicine, University of Pittsburgh, Pittsburgh, Pennsylvania, USA

^8^Research Department of Infection, University College Medical School, London, UK

^*^**Corresponding author:** ddimitro@fredhutch.org

**Technical appendix**

**1. Survey participants**

The following experts responded to our survey with quantitative estimates which have been included in the analysis:

1. Jan Albert, Department of Microbiology, Tumor and Cell Biology, Karolinska Institutet and Department of Clinical Microbiology, Karolinska University Hospital, Stockholm, Sweden
2. Charles Boucher, Department of Virology, Erasmus MC, University Medical Centre Rotterdam, Rotterdam
3. David van de Vijver , Department of Virology, Erasmus MC, University Medical Centre Rotterdam, Rotterdam
4. Christopher Hurt, Division of Infectious Diseases, Department of Medicine, University of North Carolina at Chapel Hill, USA
5. John Mellors, Chief of the Division of Infectious Diseases, School of Medicine, University of Pittsburgh, Pittsburgh, Pennsylvania, USA
6. Deenan Pillay, Head of the Research Department of Infection, University College Medical School, London, United Kingdom.

Dr. Boucher and Dr. van de Vijver have submitted a joint opinion. The responses by each virologist are labeled (V1, V2 …, V5) and presented in no particular order.

**2. Model description**


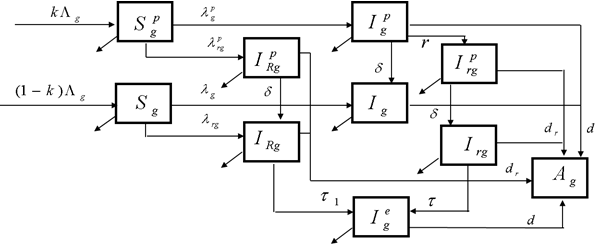


**Fig A.** Flow diagram of the extended model used in the comparison of the resistance assumptions in the responses to the virologists’ survey. Simulated population is stratified in compartments by gender as men (subscript g=m) or women (subscript g=w) and by HIV status as susceptibles (S), infected with wild HIV (I), infected with drug-resistant HIV through transmission (I_R_), individuals who developed (acquired ) resistance on PrEP (I_r_) and AIDS (A). Resistance carriers who do not use PrEP lose the ability to transmit resistance over time (superscript e) but may be at risk to fail ART when initiated. A complete description of the model including the expressions for the forces of infections (λ) is presented below.

Our model (Fig A) is implemented by a system of differential equations which govern the flows between the following population compartments:

$S_{g}^{p}$ - susceptible women (g=w) and men (g=m) using PrEP

$S_{g}$ - susceptible women (g=w) and men (g=m) not using PrEP

$I_{g}^{p}$- HIV-positive women (g=w) and men (g=m) using PrEP infected with the wild type HIV

$I_{g}$ - HIV-positive women (g=w) and men (g=m) not using PrEP infected with the wild type HIV

$I_{rg}$, $I_{rg}^{p}$ - HIV-positive women (g=w) and men (g=m) not using or using PrEP with detectable drug-resistance developed (acquired) while using PrEP (acquired resistance, ADR)

$I_{Rg},I_{Rg}^{p}$ - HIV-positive women (g=w) and men (g=m) not using or using PrEP with detectable drug-resistance transmitted to them at the time of infection (transmitted resistance, TDR)

$I_{g}^{e}$- HIV-positive women (g=w) and men (g=m) with undetectable drug-resistance after prolonged withdrawal from PrEP

$A_{g}$- women (g=w) and men (g=m) who died from AIDS

Model equations which describe the rates of change in the population compartments corresponding to one gender are:

$$\begin{matrix} \frac{dS_{g}}{dt}=(1-k)\Lambda_{g}-(\lambda_{g}+\lambda_{rg})S_{g}-\mu S_{g} \\ \frac{dS_{g}^{p}}{dt}=k\Lambda_{g}-(\lambda_{g}^{p}+\lambda_{rg}^{p})S_{g}^{p}-\mu S_{g}^{p} \\ \frac{dI_{g}}{dt}=\lambda_{g}S_{g}+\delta I_{g}^{p}-(\mu+d )I_{g} \\ \frac{dI_{Rg}}{dt}=\lambda_{rg}S_{g}+\delta I_{Rg}^{p}-\tau_{1} I_{Rg}-(\mu+d) I_{Rg} \\ \frac{dI_{rg}}{dt}=\delta I_{rg}^{p}-\tau I_{rg}-(\mu+d) I_{rg} \\ \frac{dI_{g}^{p}}{dt}=\lambda_{g}^{p}S_{g}^{p}-(rr_{a} +\delta+\mu+d) I_{g}^{p} \\ \frac{dI_{Rg}^{p}}{dt}=\lambda_{rg}^{p}S_{g}^{p}-(\delta+\mu+d) I_{Rg}^{p} \\ \frac{dI_{rg}^{p}}{dt}=rr_{a} I_{g}^{p}-(\delta+\mu+d) I_{rg}^{p} \\ \frac{dI_{g}^{e}}{dt}=\tau I_{rg}+\tau_{1} I_{Rg}-(\delta+\mu+d) I_{g}^{e} \\ \frac{dA_{g}}{dt}=d(I_{g}+I_{g}^{p})+d_{r}(I_{rg}+I_{rg}^{p}+I_{Rg}+I_{Rg}^{p}) \end{matrix}$$

where $d$and $d_{r}$are the HIV-related mortality rates for individuals infected with wild-type and resistant HIV, $\delta$is the rate at which HIV-positive users of PrEP withdraw from the product, $1/\mu$ is the average time to remain sexually active, $\tau$and $\tau_{1}$ are the rates of resistance reversion for former PrEP users who acquired drug resistance when on PrEP and for infected to whom the resistant HIV has been transmitted, *k* is the PrEP coverage, i.e, proportion of newly recruited men and women who start using PrEP, *r* is the rate of PrEP-associated resistance development assuming perfect adherence to PrEP and *r_a_* is the relative rate of resistance development due to imperfect adherence. The recruitment rate *Λ_g_* in each gender is selected to ensure population growth of 2% in absence of HIV which corresponds to demographic data from South Africa, i.e., *Λ_g_* =(*μ+0.02) N_g_* where $N_{g}=S_{g}+S_{g}^{p}+I_{g}+I_{g}^{p}+I_{rg}+I_{rg}^{p}+I_{Rg}+I_{Rg}^{p}+I_{g}^{e}$ - represent the sexually active males (g=m) or females (g=w), respectively. The biological meaning of the parameters and their values (ranges) used in the analysis are given in Table 2 in the main text.

The forces of infections ($\lambda_{g},\lambda_{rg},\lambda_{g}^{p}, and \lambda_{rg}^{p}$) by gender ($g$) are based on number of sex partners per year (*ρ_g_*), annual acquisition risk ($R_{g}^{i,j}$and $R_{rg}^{i,j}$ *)* for uninfected individual not using PrEP (i=0) or using PrEP (i=p) per partnership with infected individual not using PrEP (j=0), or using PrEP (j=p) and the fraction of the opposite gender ($\bar{g}$) which is currently in specific infectious compartment.

The subscripts r and R indicate that the infected partner is carrier of ADR and TDR respectively,

$\rho_{g}$ is the average number of partners per year for women (g=w) and men (g=m),

$\varepsilon_{z}^{xy}$ is the probability to acquire resistant HIV through transmission (given that transmission occurs) from individual with acquired (z=0) and transmitted resistance (z=1) who is PrEP user (y=p) or nonuser (y=n) to individual who is PrEP user (x=p) or non-user (x=n).

Annual risks per serodiscordent partnership in which the infected partner carries wild-type ($R_{g}^{xy}$), TDR ($R_{Rg}^{xy}$) and ADR ($R_{rg}^{xy}$*)* are derived from standard binomial models based on the number of sex acts per partnership (*n_g_*/ρ_g_), the fraction of sex acts protected by condom (c) and the HIV acquisition risk per sex act which depends on if the susceptible partner uses PrEP (x=p) or not (x=n) and if the infected partner uses PrEP (y=p) or not (y=n):

Here β_g_  is the female (g=w) and male (g=m) HIV acquisition risk per unprotected vaginal act if neither partner uses PrEP and the infected partner carry wild type HIV, β_r_ (β_R_) is the relative infectiousness of individuals with ADR (TDR) compared to infected with wild-type HIV, α_s_ (α_i_) measures the efficacy of PrEP in reducing susceptibility (infectiousness) of PrEP users, γ_r_ is the relative PrEP efficacy when exposed to drug-resistant compared to wild-type HIV, *c* is rate of condom use in general population (fraction of sex acts in which condom is used), α_c_ is the condom efficacy per act, while *r_c_* is the rate of condom replacement in PrEP users (percent reduction in condom use if PrEP is used by at least one of the partners).

PrEP interventions are initiated in populations with equal representation of the sexes (*N_m_*(0)=*N_w_*(0)) with predefined HIV prevalence in women (*P_w_)* and men (*P_m_)*. PrEP is initially prescribed to a proportion *k_1_* of the HIV-negative and to a reduced proportion of *(1-θ) k_1_* of the HIV-positive individuals. Based on that the initial size of the population compartments is set as follows:

$$\begin{matrix} S_{g}(0)=(1-k_{1})(1-P_{g})N_{g}(0) \\ S_{g}^{p}(0)=k_{1}(1-P_{g})N_{g}(0) \\ I_{g}(0)=(1-(1-\theta)k_{1})P_{g}N_{g}(0) \\ I_{g}^{p}(0)=(1-\theta)k_{1}PN_{g}(0) \\ I_{rg}^{p}(0)=I_{Rg}^{p}(0)=0 \\ I_{rg}(0)=I_{Rg}(0)=0 \end{matrix}$$

**3. Model parameterization and calibration**

**Table A Behavioral, epidemic and intervention parameters explored in the analysis**

| **Parameter** | **Description** | **Values and ranges** | | **Referen-ces** |
| --- | --- | --- | --- | --- |
|  | 1. **Behavioral and epidemic parameters (pre-intervention)** | **Prior range** | |  |
| $\beta_{w}$ | Female HIV acquisition risk per vaginal act | 0.0019 - 0.0046 | | [[1](#_ENREF_1)] |
| $\beta_{m}$ | Male HIV acquisition risk per vaginal act | (50-100%)$\beta_{w}$ | | assumed [[1](#_ENREF_1)] |
| $1/\mu$ | Average time to remain sexually active | 35 years | | [[2](#_ENREF_2), [3](#_ENREF_3)] |
| $d, d_{r}$ | HIV-related mortality rates for individuals infected with wild-type and resistant HIV | 8.3%-14.3% | | [[4](#_ENREF_4), [5](#_ENREF_5)] |
| $n_{w}, n_{m}$ | Average number of sexual acts per year for women and men | 60 - 100 | | [[6](#_ENREF_6), [7](#_ENREF_7)] |
| $\rho_{w}, \rho_{m}$ | Average number of sexual partners per year for women and men | 0.5-1.5 | | [[7](#_ENREF_7), [8](#_ENREF_8)] |
| $c$ | Rate of condom use in general population as a fraction of sex acts in which a condom is used | 20-60% | | [[6](#_ENREF_6), [7](#_ENREF_7)] |
| $\alpha_{c}$ | Condom efficacy per sex act | 0.80-0.95 | | [[9](#_ENREF_9)] |
|  | 1. **Calibration epidemic data** |  |  | |  |
| *Pw* | Initial HIV-prevalence (women) | 20% | | [[10](#_ENREF_10)] |
| *Pm* | Initial HIV-prevalence (men) | 15% | | [[10](#_ENREF_10)] |
| *Inc* | Fitted HIV-incidence (total) | 0.6-2.5% | | [[11](#_ENREF_11)] |
| *Pr_5_* | Fitted HIV-prevalence in 5 years (total) | 16.5%-18.5% | | assumed |
|  | 1. **Intervention parameters** |  | |  |
| k | PrEP coverage. Proportion of men and women who use PrEP. | 50% | | assumed |
| k_1_ | Initial fraction of the susceptibles using PrEP | 50% | | assumed |
| $\alpha_{s}$ | PrEP efficacy in reducing susceptibility per act when exposed to wild-type HIV | 90% | | assumed [[12](#_ENREF_12), [13](#_ENREF_13)] |
| $\alpha_{i}$ | PrEP efficacy in reducing infectiousness per act wild type when exposed to wild-type HIV | 90% | | assumed [[14](#_ENREF_14)] |
| γ | PrEP adherence | 14%, 50%, 100% | | assumed |
| *γ_a_* | Relative PrEP efficacy associated with poor adherence compared to perfect adherence | γ_a_= γ | | assumed[[15](#_ENREF_15), [16](#_ENREF_16)] |
| *θ* | PrEP prescription rejection rate to infected individual | 90% | | assumed |
| *δ* | Annual PrEP drop-rate by HIV-positive individuals | 1 | | assumed |
|  |  |  |  | |  |

***Model Calibration Procedure***

Demographic, behavioral and epidemiological parameters were defined and initially sampled from ranges representative of the HIV epidemics in the Sub-Saharan region (see Table A, part 1).

Next, we identified 1000 parameter sets, reflecting the HIV epidemic in South Africa using Monte Carlo filtering with the following target criteria (see Table A, part 2):

i) initial HIV prevalence of 15% and 20% among 15-49 years old men and women, respectively (Statistics South Africa Mid-year population estimates, 2010);

ii) annual incidence rate between 0.6% and 2.5% [[11](#_ENREF_11)];

iii) female incidence rate at least 30% higher than male incidence rate [[11](#_ENREF_11)] and

iv) the absolute difference in HIV prevalence over five years remains below 1% (mature epidemics).

***Pooling procedure to obtain an aggregated parameter set***

1) Triangular probability distributions have been created based on the median values and ranges suggested by the experts for each parameter (Table 2 in the main text)

2) Distributions from 1) have been aggregated by linear pooling with equal weights.

3) The aggregated parameter set, consists of ranges representing the 90% confidence level of the distributions resulting from 2).

The aggregated parameter set is used in the sensitivity analyses to study the influence of single resistance parameters or groups of related parameters on the intervention outcomes.

**4. Intervention metrics**

The following metrics are evaluated over 10 years of PrEP use in the population:

1. Resistance prevalence is measured as the prevalence of drug-resistance among HIV-positive individuals and estimated as $\frac{I_{rg}+I_{Rg}+I_{rg}^{p}+I_{Rg}^{p}}{I_{g}+I_{rg}+I_{Rg}+ I_{g}^{p}+I_{rg}^{p}+I_{Rg}^{p}+I_{g}^{e}}$
2. Transmitted resistance fraction is measured as the cumulative fraction of infections in which drug-resistant HIV is transmitted and estimated as $\frac{Number of infections in which drug-resistant HIV is transmitted}{Total number of new infections}$
3. Proportion at risk to fail ART is measured as the fraction of infected individuals with detectable or undetectable level of drug-resistance and estimated as $\frac{I_{rg}+I_{Rg}+I_{rg}^{p}+I_{Rg}^{p}+I_{g}^{e}}{I_{g}+I_{rg}+I_{Rg}+ I_{g}^{p}+I_{rg}^{p}+I_{Rg}^{p}+I_{g}^{e}}$

**5. Additional results**

The overall PrEP effectiveness is measured by two metrics by comparing scenarios with and without PrEP:

- cumulative fraction of infections prevented (CPF) due to PrEP use over 10 years which is estimated as:

$1-\frac{Cumulative number of infections (with PrEP)}{Cumulative number of infections (no PrEP)}$ .

- reduction of HIV prevalence due to PrEP use after 10 years which is estimated as $1-\frac{HIV prevalence (with PrEP)}{HIV prevalence (no PrEP)}$.


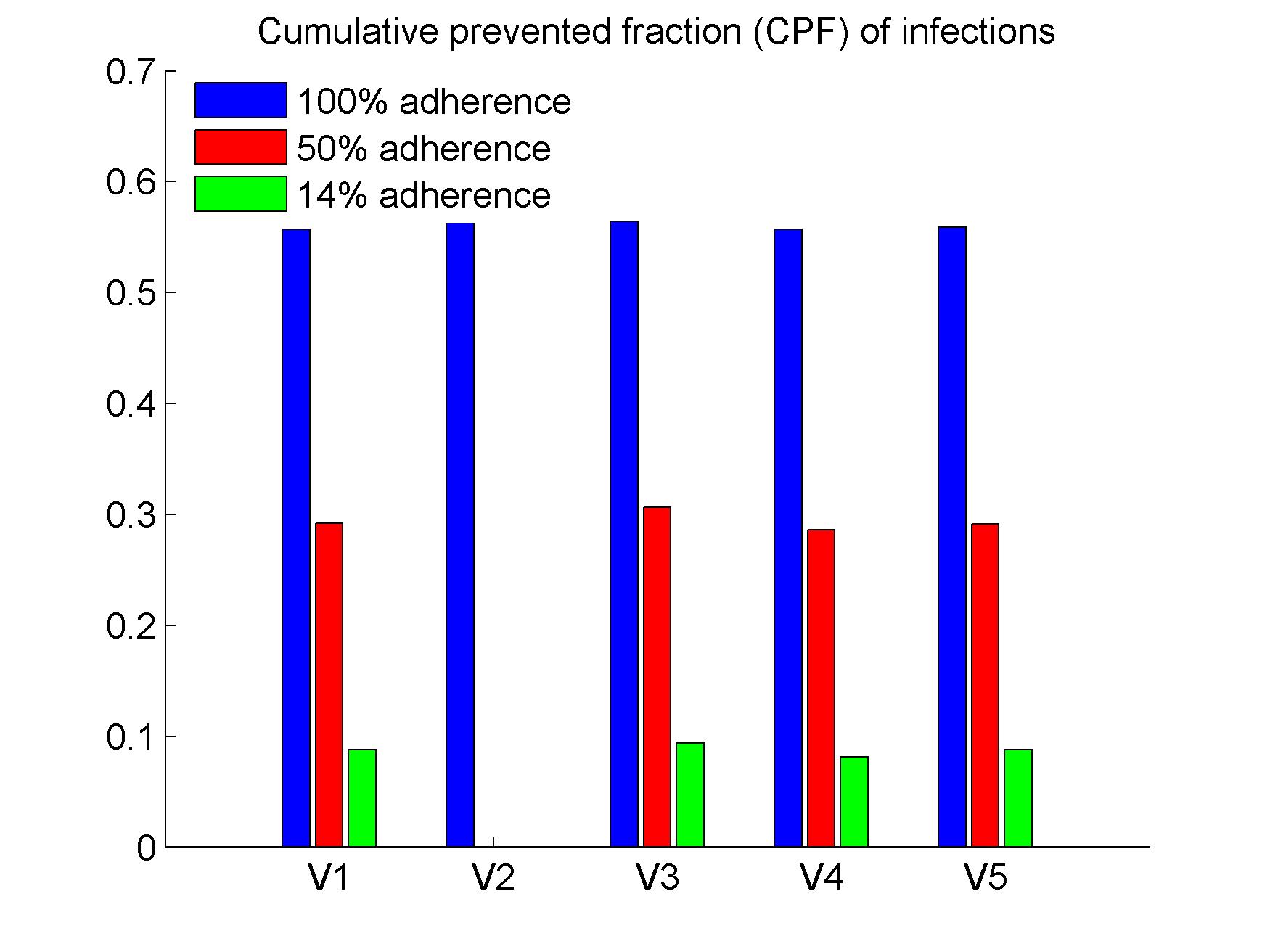

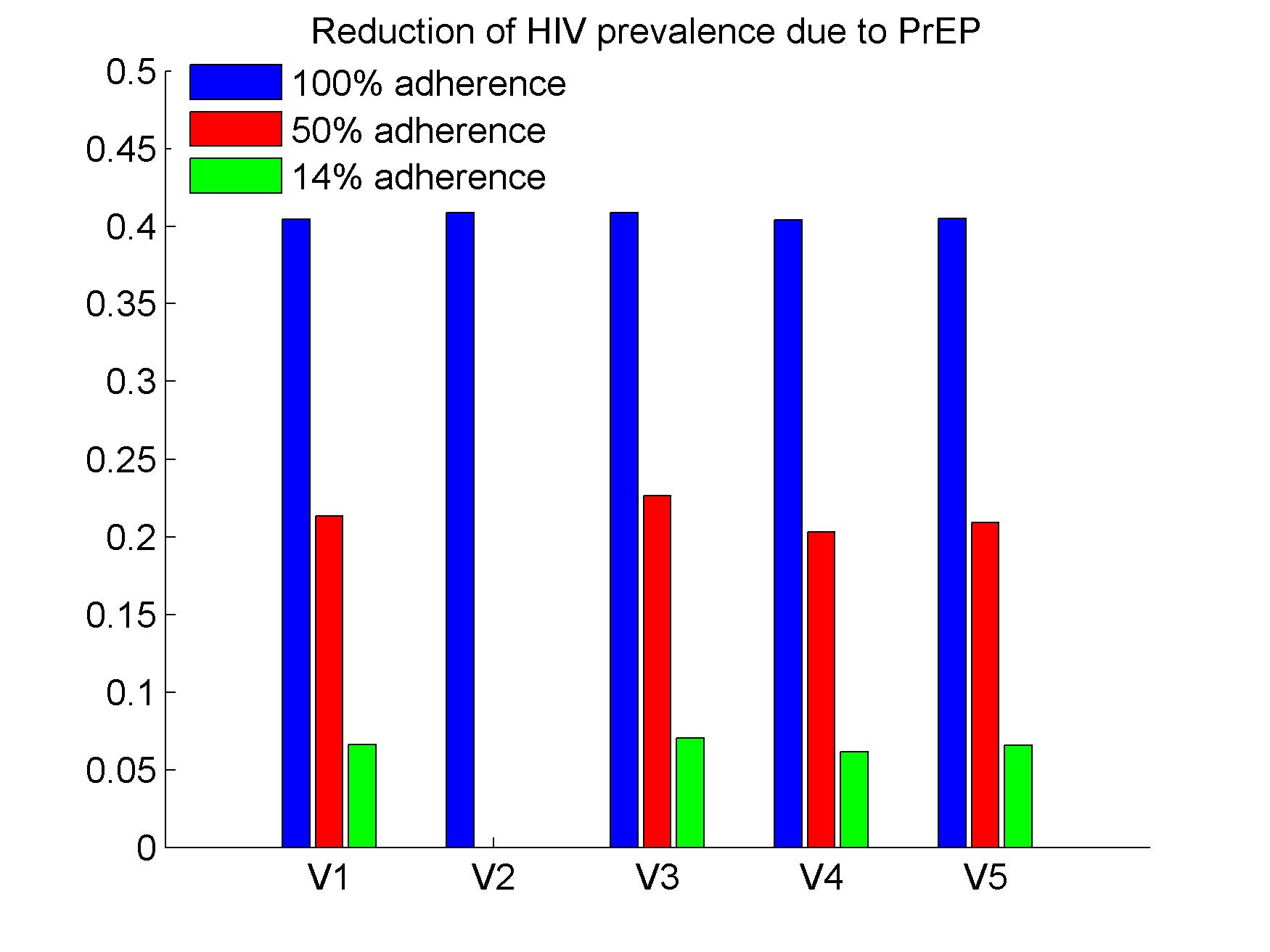


A)

B)

Fig B. Projected impact of 10 years of PrEP use on: A) the cumulative number of infections and B) the HIV prevalence when the model is parameterized with the responses of the participants in the virologists survey assuming different levels of adherence to PrEP. The bars represent the mean metrics estimates based on 1,000 epidemics simulated. Intervention parameters are fixed on their baseline values from Table A, part 3.

The overall PrEP effectiveness in terms of CPF is not substantially impacted by the assumptions related to resistance. The variation in CPF is primarily due to the assumed relative infectiousness of resistance carriers (positive correlation) and the relative PrEP efficacy against resistant HIV (negative correlation) which account for 51% and 43% of the variance of CPF. These two factors have been already identified as key drivers of the influence of resistance on the PrEP effectiveness in published studies. [[17](#_ENREF_17)] Similarly, the reduction of HIV prevalence shows no significant variation across resistance related parameter sets.

**6. Sensitivity Analysis**

**6.1 Univariate sensitivity analysis**


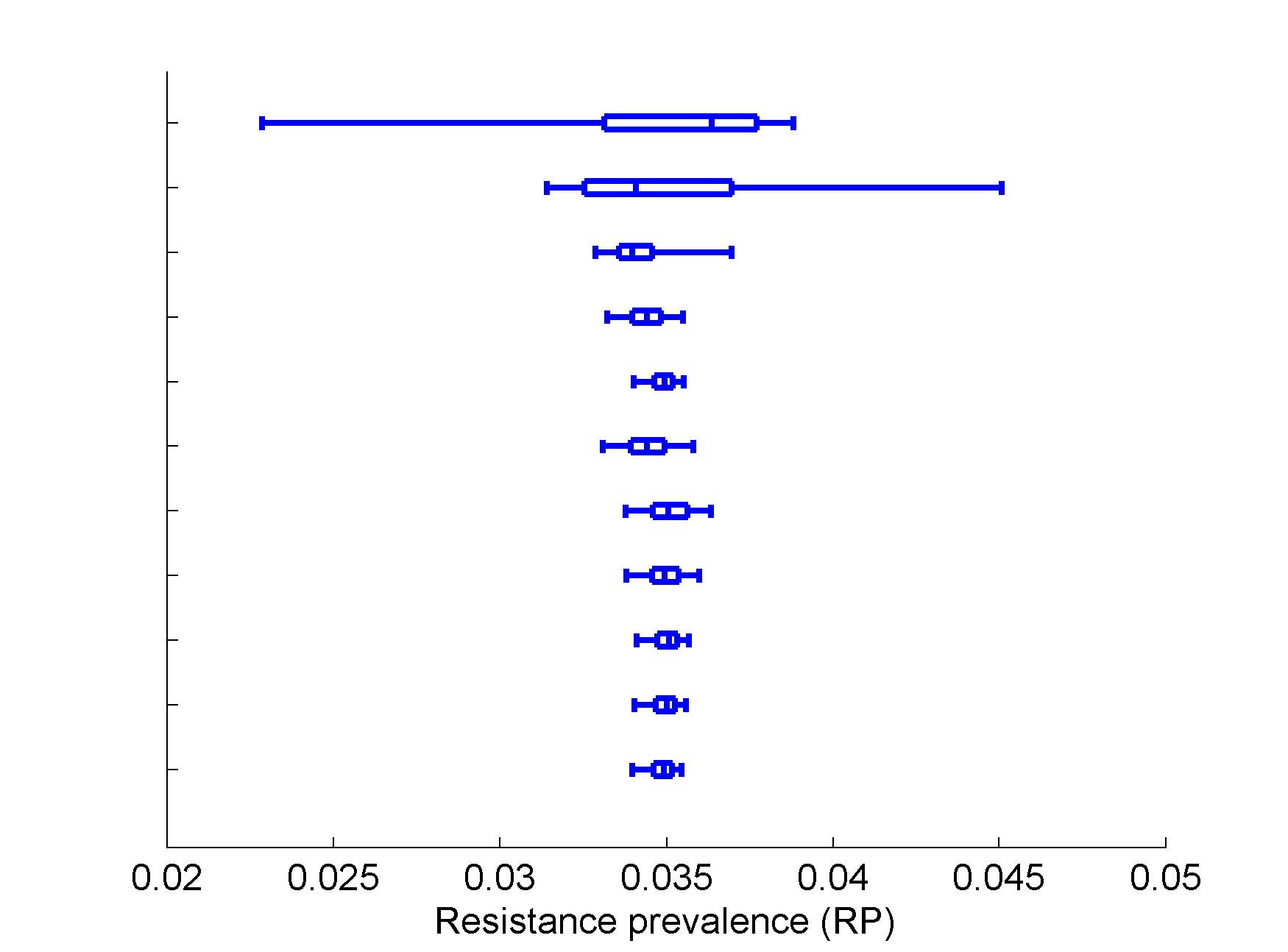

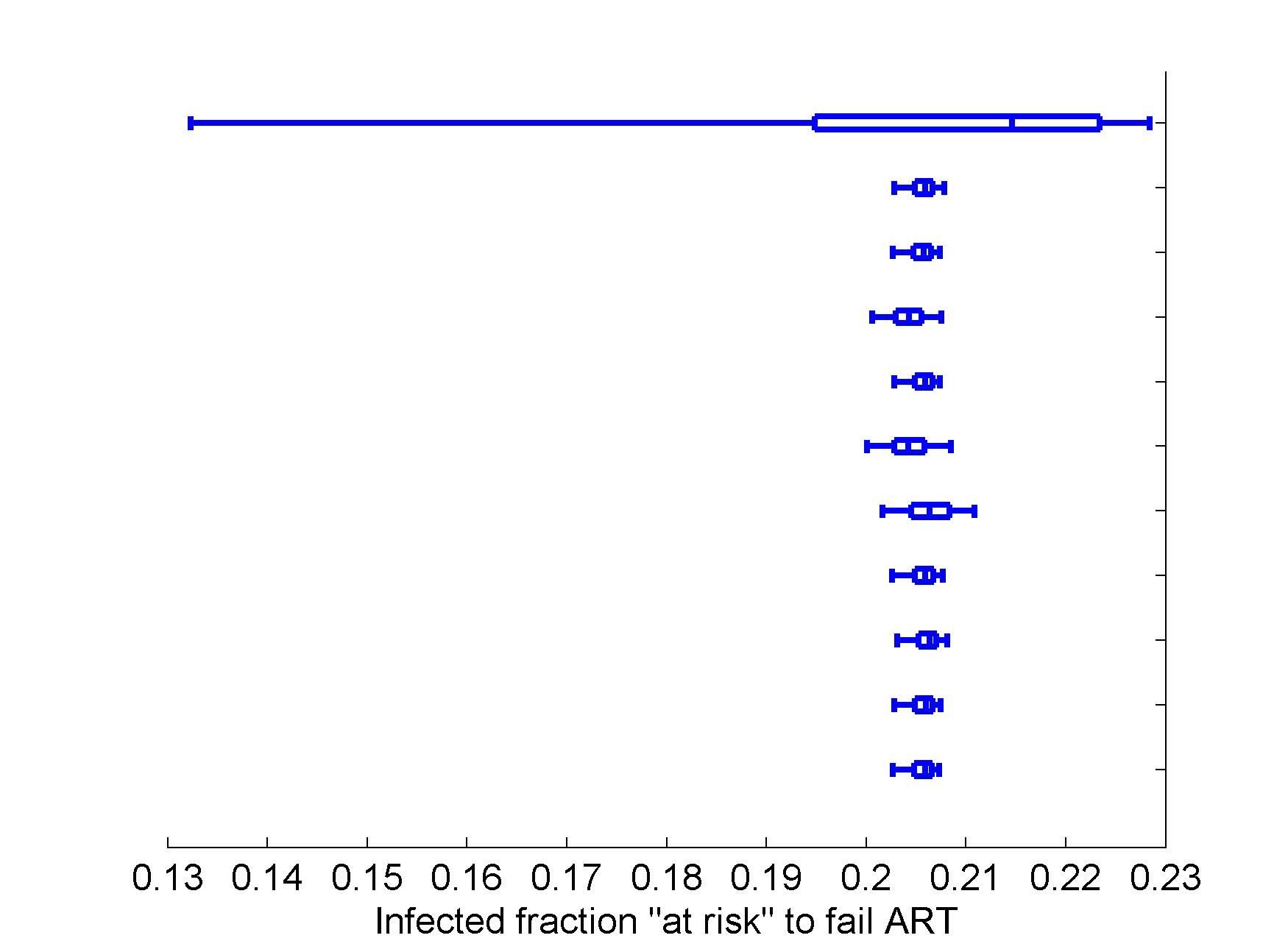

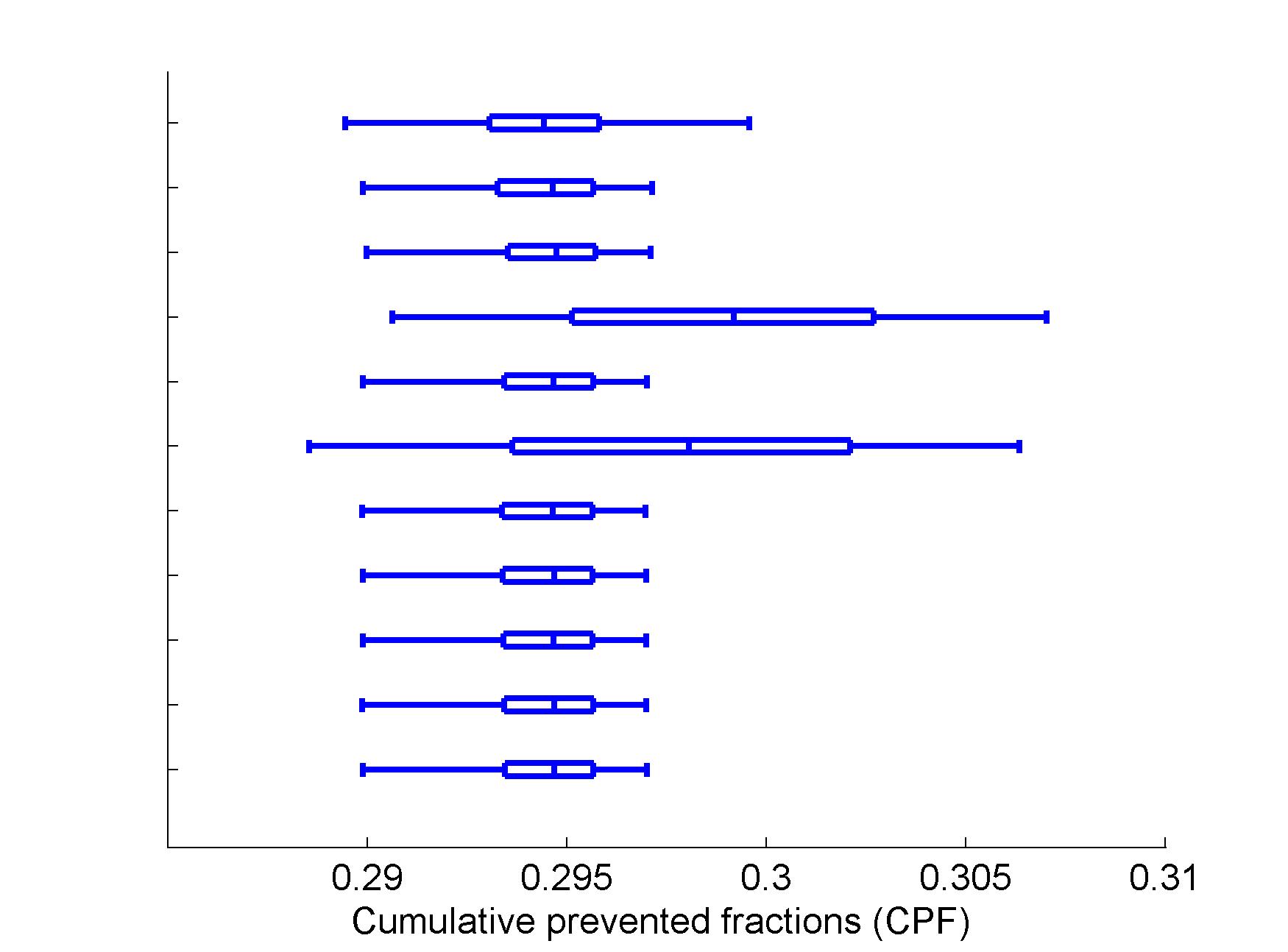

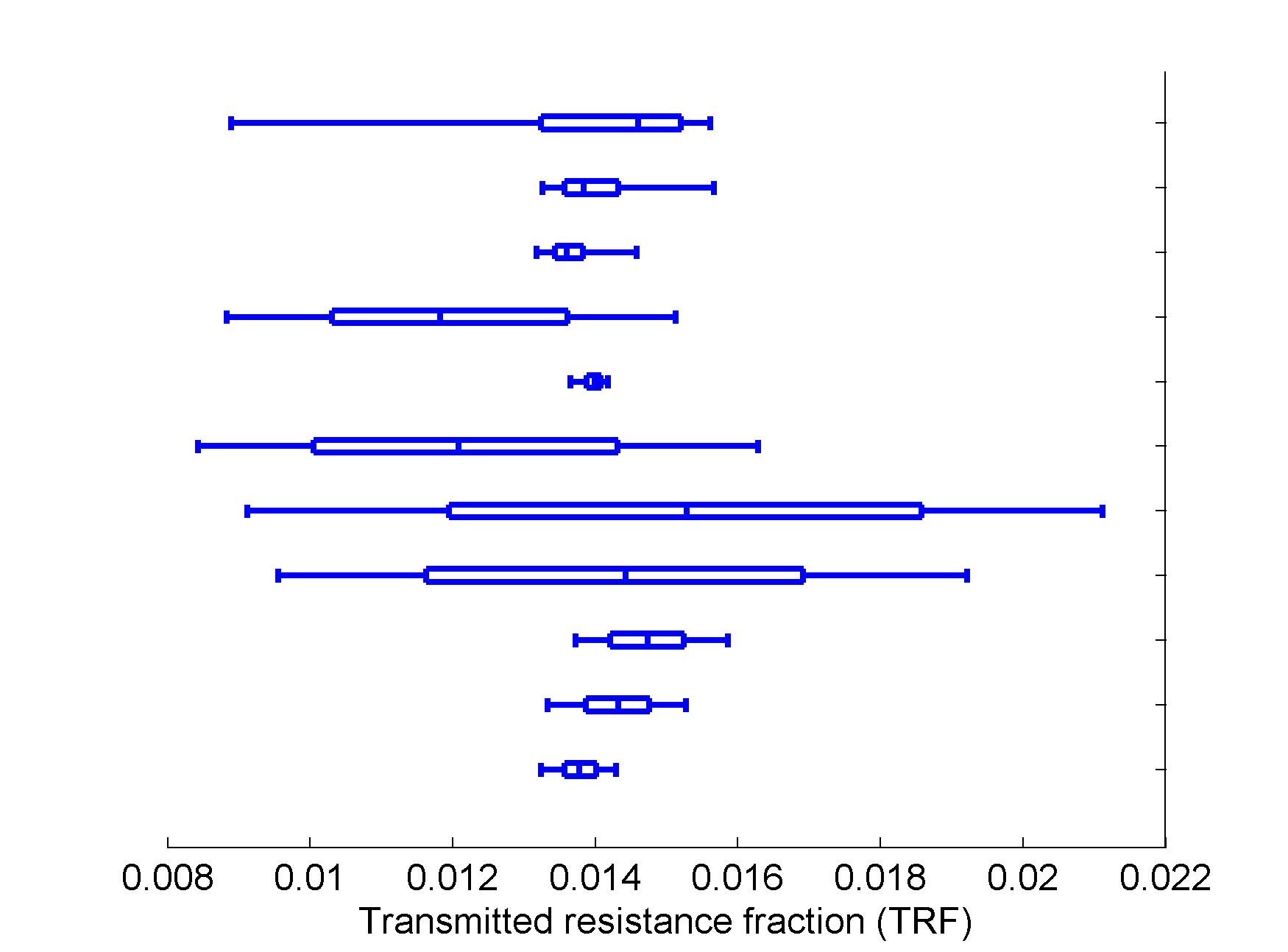


Parameter varied:

ADR emergence rate

Reversion rate ADR

Reversion rate TDR

Infectiousness ADR

Infectiousness TDR

Relative eff. against res.

ADR transm: on→off

ADR transm: on→on

ADR transm: off→off

ADR transm: off→on

TDR transm: off→off

Parameter varied:

ADR emergence rate

Reversion rate ADR

Reversion rate TDR

Infectiousness ADR

Infectiousness TDR

Relative eff. against res.

ADR transm: on→off

ADR transm: on→on

ADR transm: off→off

ADR transm: off→on

TDR transm: off→off

A)

B)

C)

D)

Fig C. Projected impact of single resistance assumption on A) the cumulative fraction of prevented infections (CPF); B) the cumulative fraction of infections in which resistance is transmitted; C) the resistance prevalence due to PrEP; and D) the fraction of infected individuals who have shown resistance at some time point over 10 years of PrEP use. When varied resistance parameters are sampled from their pooled ranges based on the responses to the virologists survey (Table 2 in the main text). When fixed resistance parameters take their mean values from the pooled analysis. The boxplots (median, 2.5th, 25th, 75th, 97.5th percentiles) reflect the variation in impact estimates based on 1,000 epidemics simulated assuming 50% adherence and medium (θ=0.9, δ=1) control on the access of infected individuals to PrEP is assumed.

**6.2 Multivariate sensitivity analysis**

We study the uncertainty in the intervention metrics generated by the individual resistance factors in multivariate sensitivity analyses. All the parameters are varied across wider ranges aggregated from the survey responses but without taking each set of parameter estimates together. Positive and negative correlation of the ADR emergence rate with adherence are explored. Partial rank correlation coefficients (PRCC) between the input and output parameters have been calculatedto evaluate the impact of single parameters on the monotonicity of the outcomes, i.e., if the increase in the input is correlated with an increase or decrease in the outcome (see Fig 4 in the main text). Model-independent variance-based methods are used to partition the outcomes variance into fractions which can be explained by variation in input parameters (individually and in interactions with other parameters). (see Saltelli, 2000) First-order Sobol indeces measure the main effect that each input parameter has on the output as a fractional contribution to the output variance. Total effect Sobol indeces measure the effect that each input parameter has including the interactions effects with other inputs as an estimate of the total contribution of the parameter to the output variation.

**Table B. Variance decomposition of the intervention metrics**

First-order Sobol indices:

| Parameter\Outcome | RP | at risk | CPF | TRF |
| --- | --- | --- | --- | --- |
| **ADR emergence rate** | 0.552646 | 0.989544 | * | 0.1045 |
| **Reversion rate ADR** | 0.404985 | * | * | 0.038519 |
| **Reversion rate TDR** | 0.019292 | * | * | * |
| **Infectiousness ADR** | * | * | 0.43196 | 0.134384 |
| **Infectiousness TDR** | * | * | * | * |
| **Relative eff. against res.** | * | * | 0.513962 | 0.190211 |
| **ADR transm: on→off** | * | * | * | 0.296008 |
| **ADR transm: on→on** | * | * | * | 0.161478 |
| **ADR transm: off→off** | * | * | * | 0.012092 |
| **ADR transm: off→on** | * | * | * | * |
| **TDR transm: off→off** | * | * | * | * |

Total effect Sobol indices:

| Parameter\Outcome | RP | at risk | CPF | TRF |
| --- | --- | --- | --- | --- |
| **ADR emergence rate** | 0.560367 | 0.989580 | 0.031001 | 0.119470 |
| **Reversion rate ADR** | 0.411909 | * | 0.012810 | 0.045084 |
| **Reversion rate TDR** | 0.023216 | * | * | * |
| **Infectiousness ADR** | * | * | 0.465786 | 0.152552 |
| **Infectiousness TDR** | * | * | * | * |
| **Relative eff. against res.** | * | * | 0.542550 | 0.215935 |
| **ADR transm: on→off** | * | * | * | 0.314025 |
| **ADR transm: on→on** | * | * | * | 0.179532 |
| **ADR transm: off→off** | * | * | * | 0.015080 |
| **ADR transm: off→on** | * | * | * | * |
| **TDR transm: off→off** | * | * | * | * |

Legend: * - less than 1%, green – 1%-5%, black – above 5%, red – most influential

**7. Alternative scenarios**

Assumptions regarding adherence and controlled access to PrEP by infected individuals do not directly concern drug resistance. In real interventions the levels of the adherence and access control will depend on the specific implementation and the product acceptibility in the particular populations. Questions regarding adherence and access control carry no virological uncertainty and as such were not included in our survey. However, in the modeling studies these assumptions have a strong influence on the intervention outcomes related to resistance since they affect the likelihood of breakthrough infection, initiation and duration of PrEP use by infected individuals. Moreover, these assumptions control the importance of other resistance assumptions and can not be ignored when the overall impact of resistance is evaluated.

**7.1 Impact of overall adherence level and controlled access to PrEP**

We analyze the influence of the adherence and access control on the intervention outcomes under 9 different scenarios with fixed adherence at 3 levels – low (25%), medium (50%) and high (75%) and access control at 3 levels:

- weak, in which 20% of the infected individuals may have an access to PrEP (θ=0.8) and people who acquire HIV when on PrEP continue to take PrEP for and average of 2 years (δ=0.5);
- medium, in which 10% of the infected individuals may have an access to PrEP (θ=0.9) and people who acquire HIV when on PrEP continue to take PrEP for and average of 1 years ( δ=1);
- strong, in which infected individuals have no access to PrEP (θ=1) and people who acquire HIV when on PrEP continue to take PrEP for and average of 6 months (δ=2)

while other resistance parameters are varied independently.


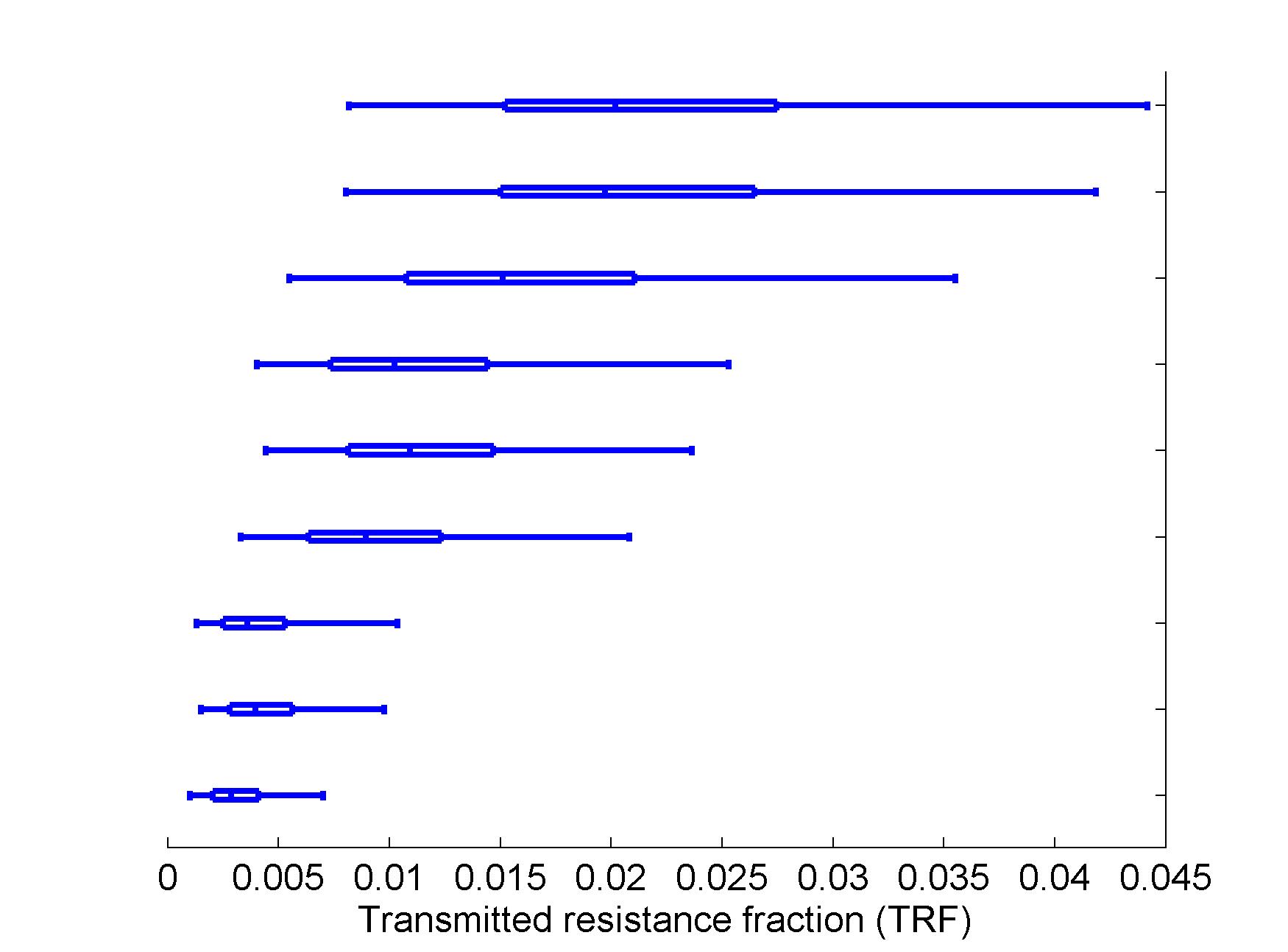

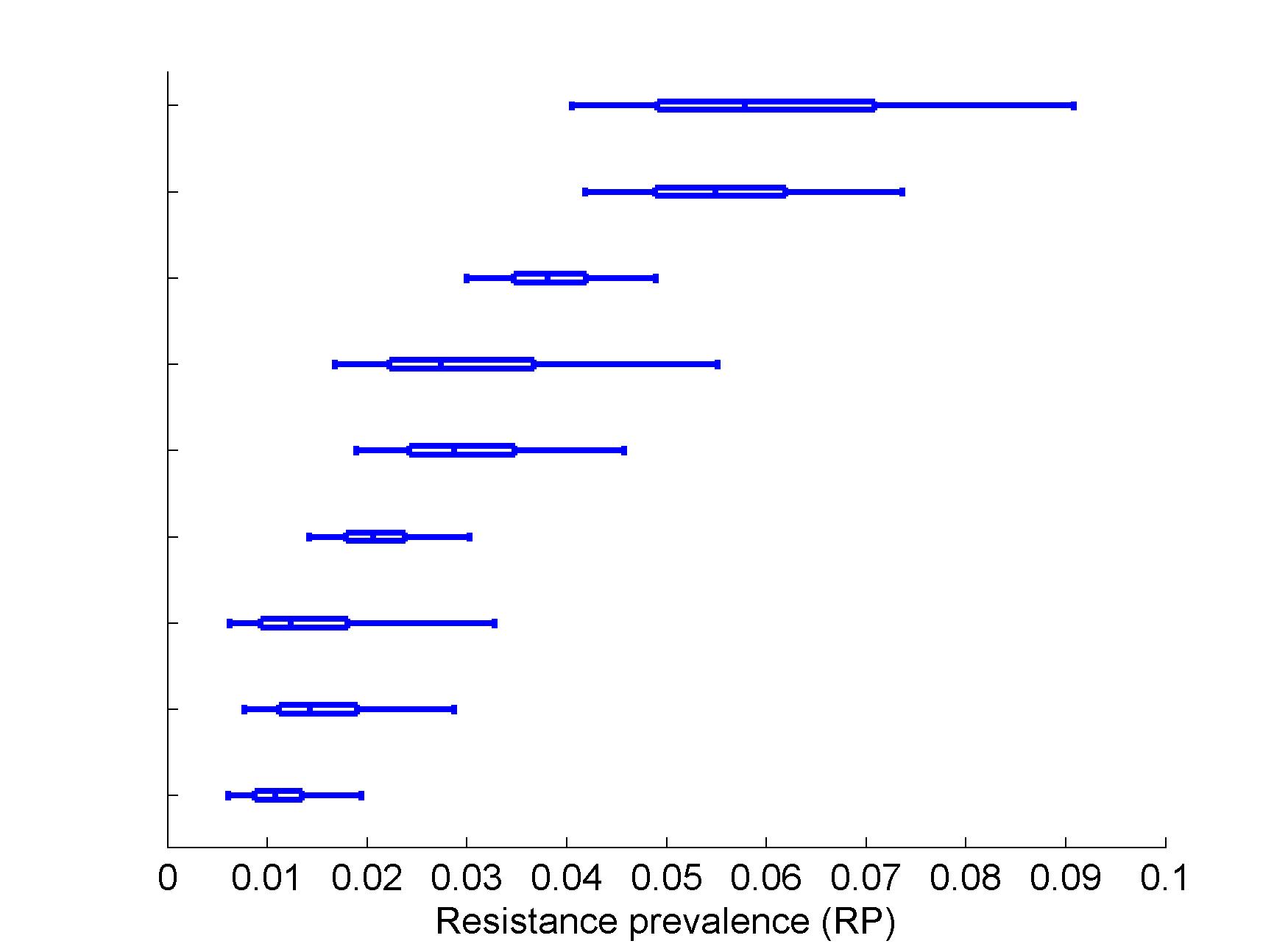

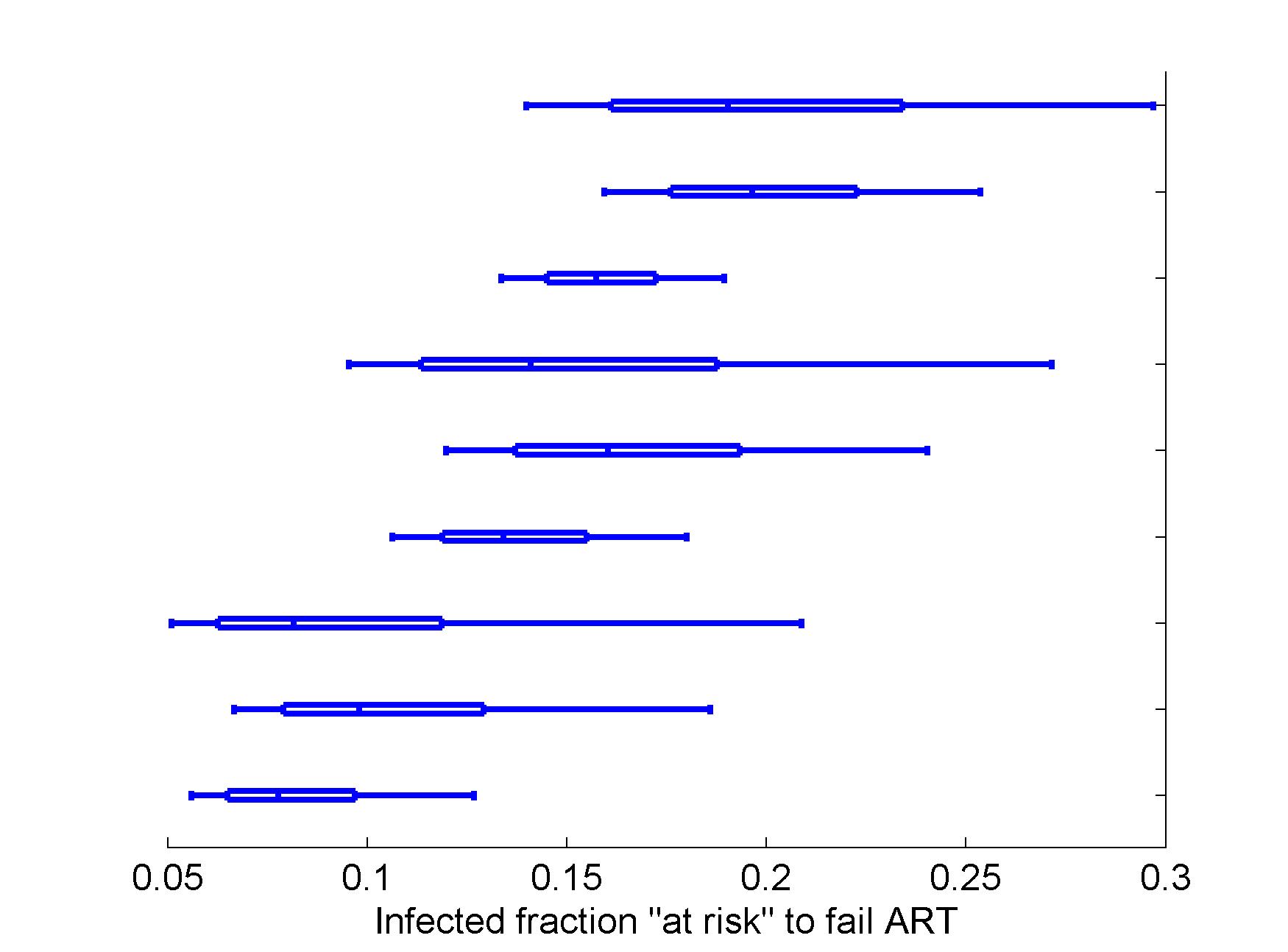

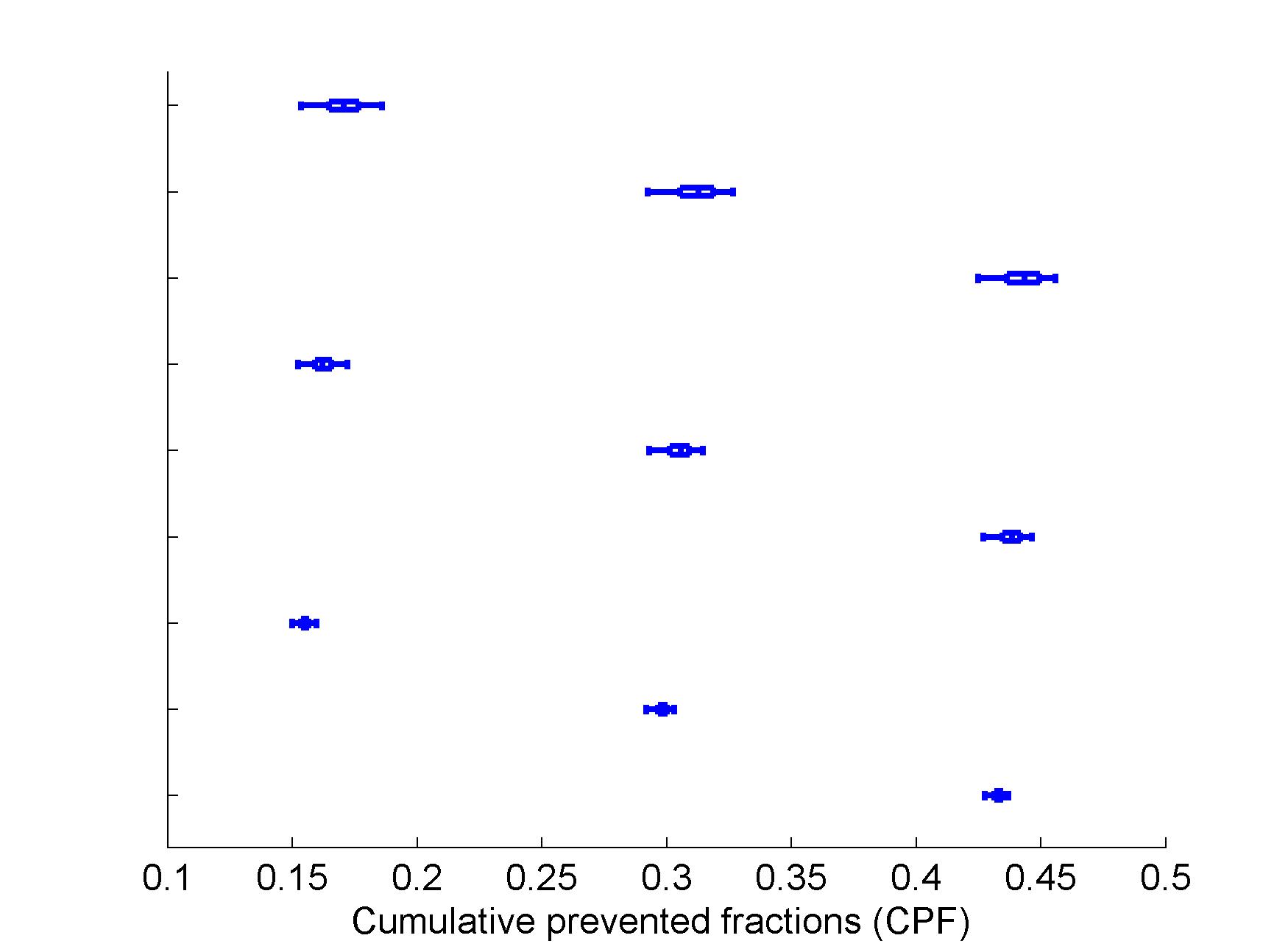


Scenarios:

25% adh, weak control

50% adh, weak control

75% adh, weak control

25% adh, medium control

50% adh, medium control

75% adh, medium control

25% adh, strong control

50% adh, strong control

75% adh, strong control

A)

B)

C)

D)

Scenarios:

25% adh, weak control

50% adh, weak control

75% adh, weak control

25% adh, medium control

50% adh, medium control

75% adh, medium control

25% adh, strong control

50% adh, strong control

75% adh, strong control

Fig D. Projected impact of resistance assumptions on A) the resistance prevalence due to PrEP; B) the fraction of infected individuals “at risk” to fail ART; C) the cumulative fraction of prevented infections (CPF) and D) the cumulative fraction of infections in which resistance is transmitted over 10 years of PrEP use. All resistance parameters are sampled from their pooled ranges based on the responses to the virologists survey (Table 2 in the main text). The boxplots (median, 2.5th, 25th, 75th, 97.5th percentiles) reflect the variation in impact estimates based on 1,000 epidemics simulated. Levels of adherence explored are low (25%), medium (50%) and high (75%). The access control is weak (θ=0.8, δ=0.5), medium (θ=0.9, δ=1) or strong (θ=1, δ=2). The scenario analyzed and presented in the main text is highlighted.

Fig D presents the projected impact of resistance under 9 different scenarios regarding adherence and restricted access of infected individuals to PrEP with all resistance parameters varied in pooled ranges based on the survey responses (see Table 2 in the main text). It shows that adherence is key determinant of the expected fraction of infection prevented with a median 10-year CPF increasing from 15% to above 40% when adherence improves from 25% to 75% (panel C). Controled access to PrEP is more important for the resistance related outcomes of the intervention (infections with transmitted resistance, resistance prevalence and fraction “at risk” to fail ART). The median fraction of TDR declines from 2% assuming weak control to below 0.5% with strong control of PrEP usage (panel D). Similarly, the projected prevalence of resistance among infected individuals drops from 6% to below 2% and the proportion with elevated risk to fail ART decreases from 20% to 8-9%. This analysis suggests that the resistance due to PrEP could be effectively controlled by more stringent procedures of initial and periodic HIV testing of PrEP users. If the guidance of the Truvada label which recommends HIV testing every 3 months is properly followed, the number of infected people using PrEP long enough to develop resistance will be reduced significantly and the influence of the biological assumptions discussed in this study will be diminished.

**7.2 Analysis of the relationship between PrEP efficacy and PrEP adherence**


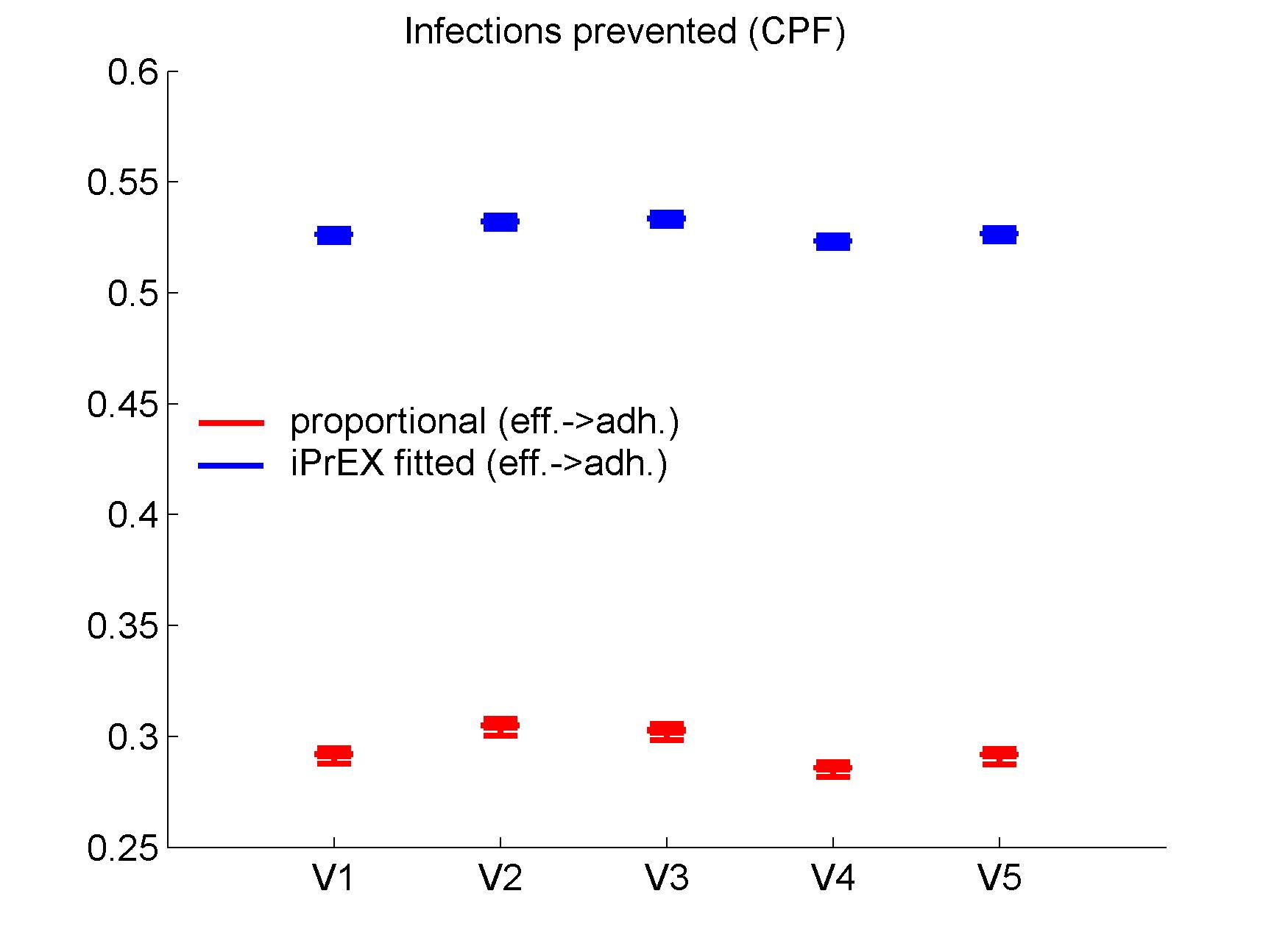

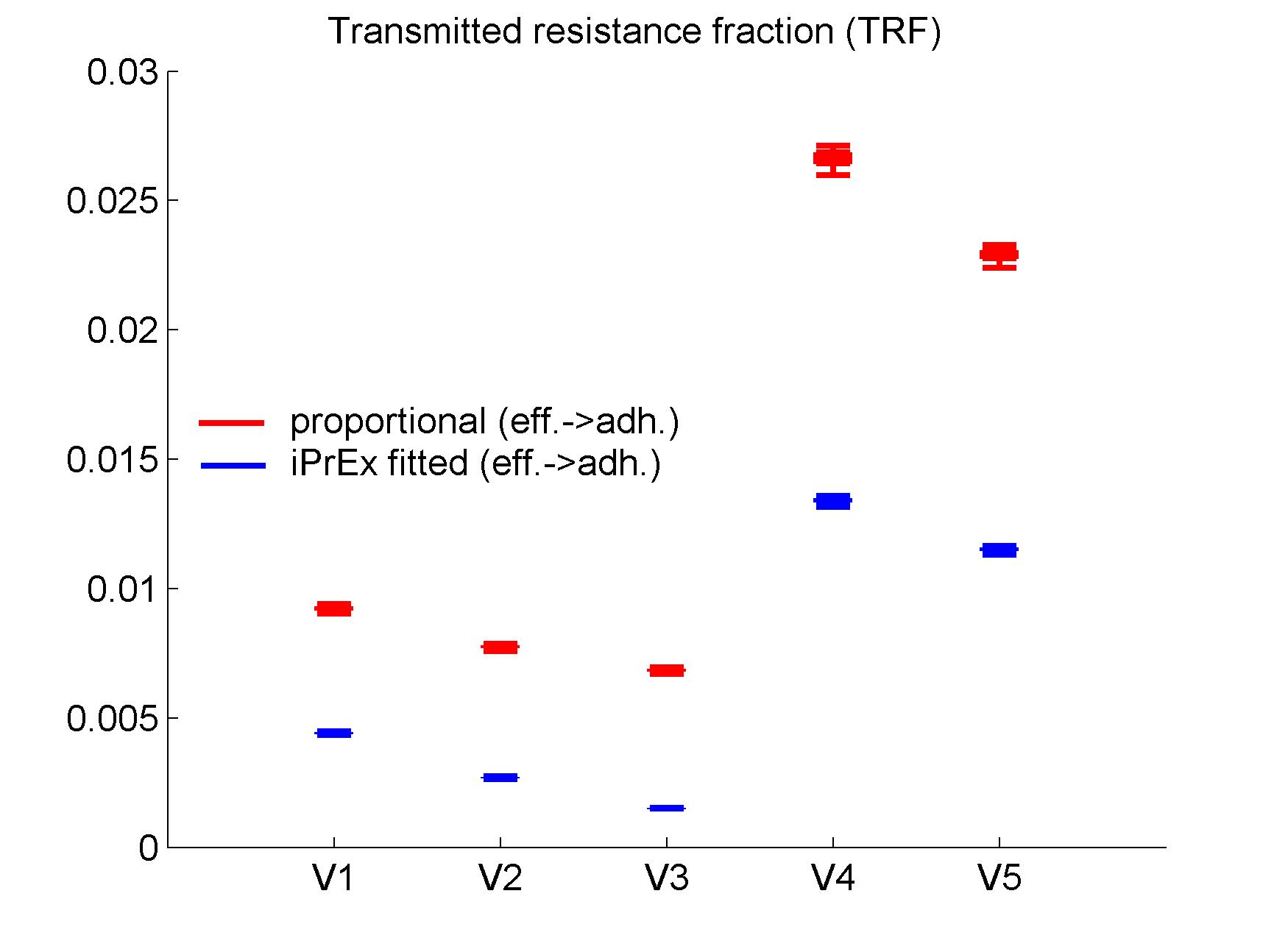

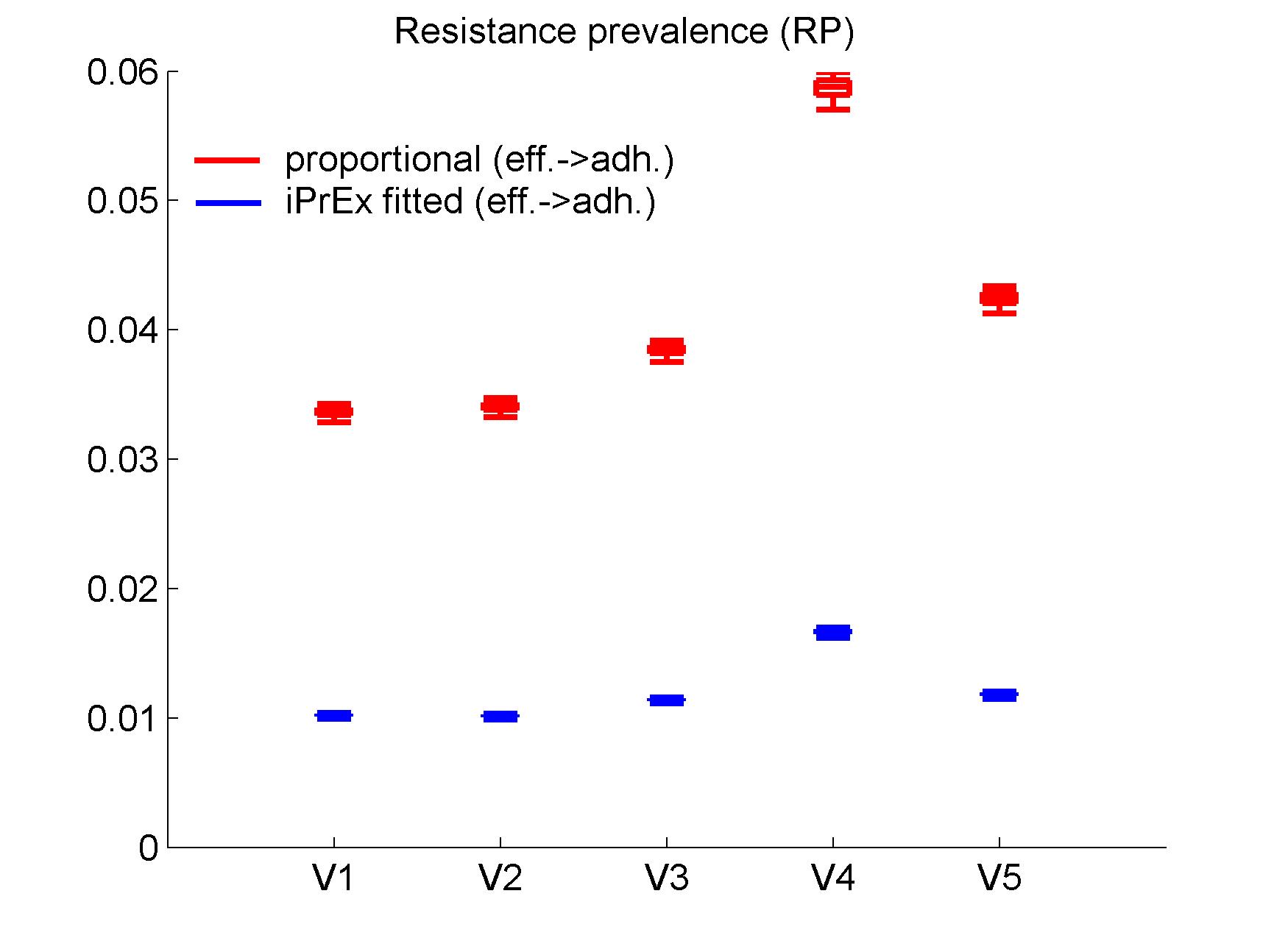


A)

B)

C)

D)


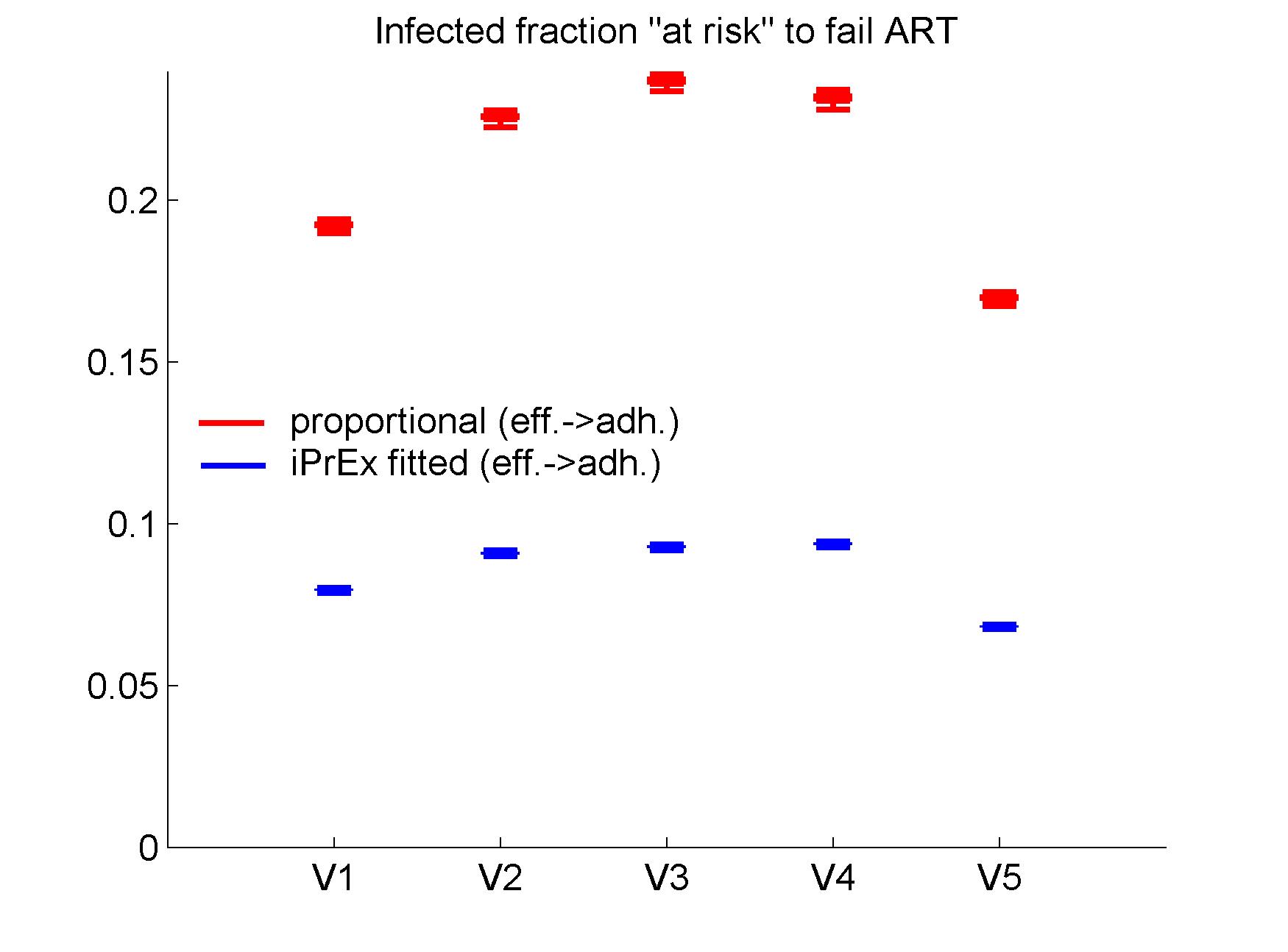


Fig E Comparison of the cumulative fraction of prevented infections (CPF), resistance prevalence due to PrEP (RP), cumulative fraction of infections in which resistance is transmitted (TRF) and the “at risk” fraction of infected individuals projected over 10 years of PrEP use on when PrEP efficacy per act is assumed proportional to adherence (red) or fitted to efficacy estimates presented by the iPrEx team in [[13](#_ENREF_13)](blue) . The model is parameterized with the responses of the virologists. The boxplots (median, 2.5th, 25th, 75th, 97.5th percentiles) reflect the variation in impact estimates based on 1,000 epidemic parameters sets used. Intervention parameters are fixed on their baseline values from Table A, part 3.

Comparison between scenarios employing different correlations between efficacy and adherence shows that improved PrEP efficacy with imperfect adherence (blue) is associated with lower resistance metrics (resistance prevalence, transmitted resistance fraction, fraction at risk to fail ART) and with increase in the effectiveness of the PrEP intervention.

**References:**

**1. Boily, M.C., et al., *Heterosexual risk of HIV-1 infection per sexual act: systematic review and meta-analysis of observational studies.* Lancet Infectious Diseases, 2009. 9(2): p. 118-129.**

**2. UNAIDS/WHO *AIDS epidemic update: November 2009* 2009.**

**3. Ferry, B., et al., *Comparison of key parameters of sexual behaviour in four African urban populations with different levels of HIV infection.* AIDS, 2001. 15: p. S41-S50.**

**4. Morgan, D., et al., *HIV-1 infection in rural Africa: is there a difference in median time to AIDS and survival compared with that in industrialized countries?* AIDS, 2002. 16(4): p. 597-603.**

**5. Porter, K. and B. Zaba, *The empirical evidence for the impact of HIV on adult mortality in the developing world: data from serological studies.* AIDS, 2004. 18: p. S9-S17.**

**6. Johnson, L., et al., *Sexual behaviour patterns in South Africa and their association with the spread of HIV: insights from a mathematical model.* Demographic Research, 2009. 21(11): p. 289-340.**

**7. Kalichman, S.C., et al., *Heterosexual anal intercourse among community and clinical settings in Cape Town, South Africa.* Sexually Transmitted Infections, 2009. 85(6): p. 411-415.**

**8. Todd, J., et al., *Reported number of sexual partners: comparison of data from four African longitudinal studies.* Sexually Transmitted Infections, 2009. 85(Suppl 1): p. i72-i80.**

**9. Foss, A.M., et al., *A systematic review of published evidence on intervention impact on condom use in sub-Saharan Africa and Asia.* Sexually Transmitted Infections, 2007. 83(7): p. 510-516.**

**10. Statistics South Africa *Mid-year population estimates*. 2010.**

**11. Rehle, T.M., et al., *A Decline in New HIV Infections in South Africa: Estimating HIV Incidence from Three National HIV Surveys in 2002, 2005 and 2008.* Plos One, 2010. 5(6): p. e11094.**

**12. Baeten, J.M., et al., *Antiretroviral Prophylaxis for HIV Prevention in Heterosexual Men and Women.* New England Journal of Medicine, 2012. 367(5): p. 399-410.**

**13. Anderson, P.L., et al., *Emtricitabine-Tenofovir Concentrations and Pre-Exposure Prophylaxis Efficacy in Men Who Have Sex with Men.* Science Translational Medicine, 2012. 4(151): p. 151ra125.**

**14. Prada, N., et al., *Drug-susceptible HIV-1 infection despite intermittent fixed-dose combination tenofovir/emtricitabine as prophylaxis is associated with low-level viremia, delayed seroconversion, and an attenuated clinical course.* Jaids-Journal Of Acquired Immune Deficiency Syndromes, 2008. 49(2): p. 117-122.**

**15. Abbas, U.L., et al., *Factors Influencing the Emergence and Spread of HIV Drug Resistance Arising from Rollout of Antiretroviral Pre-Exposure Prophylaxis (PrEP).* Plos One, 2011. 6(4): p. e18165.**

**16. Hallett, T.B., et al., *Optimal Uses of Antiretrovirals for Prevention in HIV-1 Serodiscordant Heterosexual Couples in South Africa: A Modelling Study.* Plos Medicine, 2011. 8(11): p. e1001123.**

**17. Dimitrov, D., et al., *Analytic Review of Modeling Studies of ARV Based PrEP Interventions Reveals Strong Influence of Drug-Resistance Assumptions on the Population-Level Effectiveness.* PLoS ONE, 2013. 8(11): p. e80927-.**
