# Supplementary material for: How Much Do We Know about Drug Resistance Due to PrEP Use? Analysis of Experts’ Opinion and Its Influence on the Projected Public Health Impact
Source: PLoS One. 2016 Jul 8;11(7):e0158620. doi: 10.1371/journal.pone.0158620 (PMC4938235; doi:10.1371/journal.pone.0158620)
Supplement: S2 Text — Template of the questionnaire on emergence, persistence, and transmission of HIV drug resistance due to PrEP use used in the study. (PDF) [file pone.0158620.s002.pdf]

Polling Information On Key Parameters That Determine the Potential for the Spread of Drug Resistant HIV Due to PrEP

Dobromir Dimitrov, Marie-Claude Boily, Timothy Hallett , David van de Vijver

Introduction

The following questions are put together to solicit opinion from expert virologists. Please indicate your answers quantitatively and provide comments which may give rational for your answers. The aim is to determine areas of agreement and uncertainty and to assist the parametrisation of mathematical models.

Definitions

The focus here is specifically on PrEP being a daily oral dose of TDF or TDF/FTC, and ART being TDF-based. Please indicate if you use data that do not correspond to these definitions.

Instructions

For each question there is a line with a scale and we ask for an indication of where on that line the true value lies (location and uncertainty). In the example below, a question is asked about the rate ratio of an event and a scale is provided ***(note, a logarithmic scale)****; the respondent has indicated (in red) that the rate is usually less than 1.0, but not usually more than 0.3 and with a typical value of approximately 0.5*

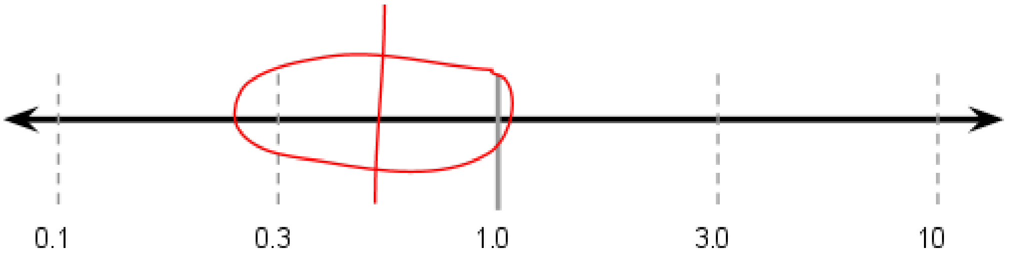

Please provide comments which may give rational for your answers (optional)

1. Rate of resistance development for HIV+ PrEP users.

a) What is the average time it takes for resistance to develop (to any detectable level) in users who acquire HIV and subsequently use PrEP with perfect compliance?

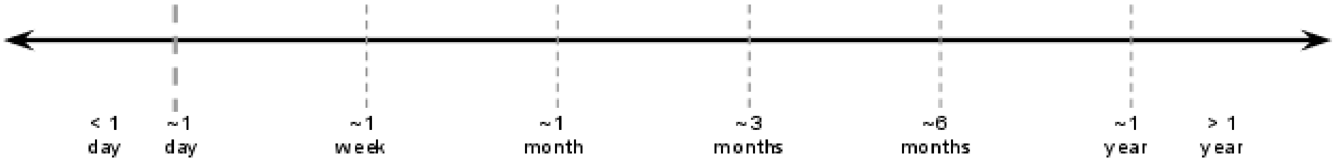

b) What is the average time it takes for resistance to develop (to any detectable level) in users who acquire HIV and subsequently use PrEP with intermittent compliance (missing doses every other day).

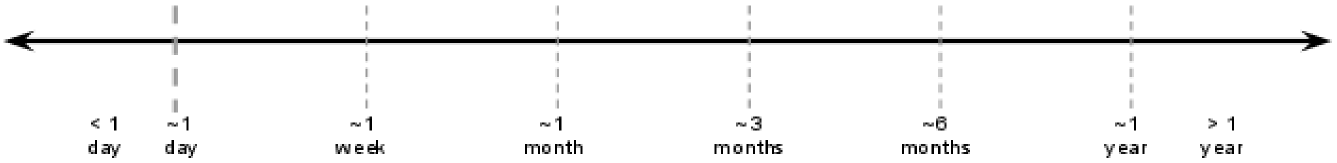

c) What is the average time it takes for resistance to develop (to any detectable level) in users who acquire HIV and subsequently use PrEP with low compliance (taking only 1 dose per week).

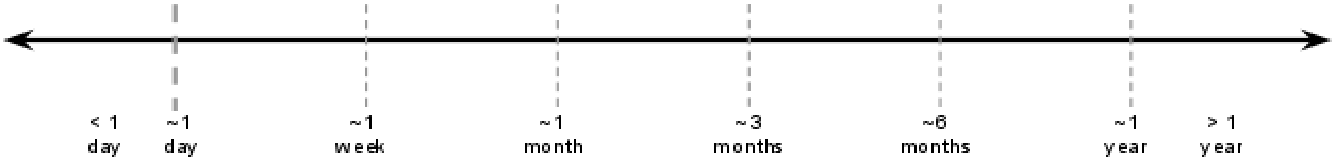

NB. These guidelines are not drawn to scale.

Comments:

2. Transmission from an individual with acquired drug-resistant HIV

a) For an individual that had acquired drug-resistance whilst on PrEP but who now has not used PrEP for <u>one week</u>, what is the *relative chance* of transmitting a resistant virus compared to a wild-type virus (given that infection has occured).

i. … to someone who is not on PrEP

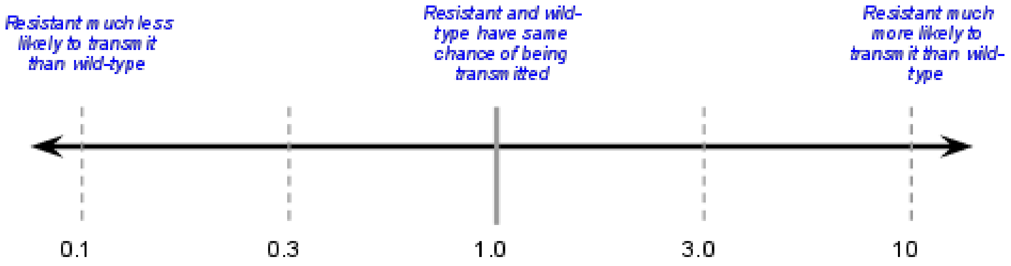

ii. … to someone who is currently taking PrEP (and in good compliance)

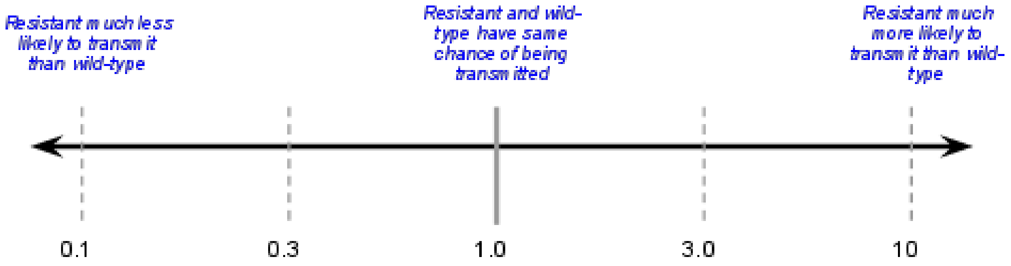

b) For an individual that acquire drug-resistance whilst on PrEP but who now has not been using PrEP for <u>one year</u>, what is the *relative chance* of transmitting a resistant virus compared to a wild-type virus (given that infection has occured) .
Note: This could change after one year due to reversion

i. … to someone who is not on PrEP

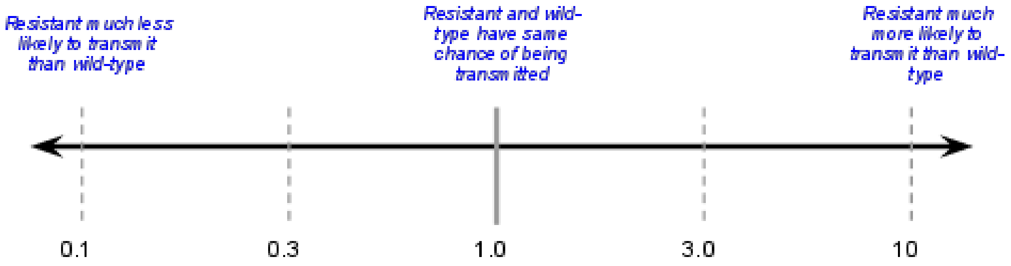

ii. … to someone who is currently taking PrEP (and in good compliance)

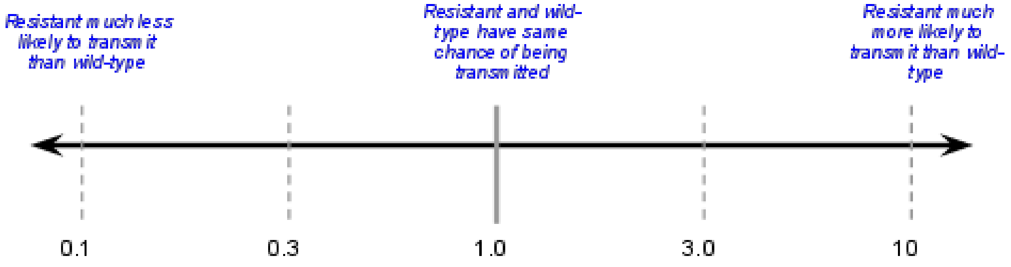

c) What is the level of protection of PrEP against drug-resistant HIV (Assumed level of protection against wild type HIV is provided.)

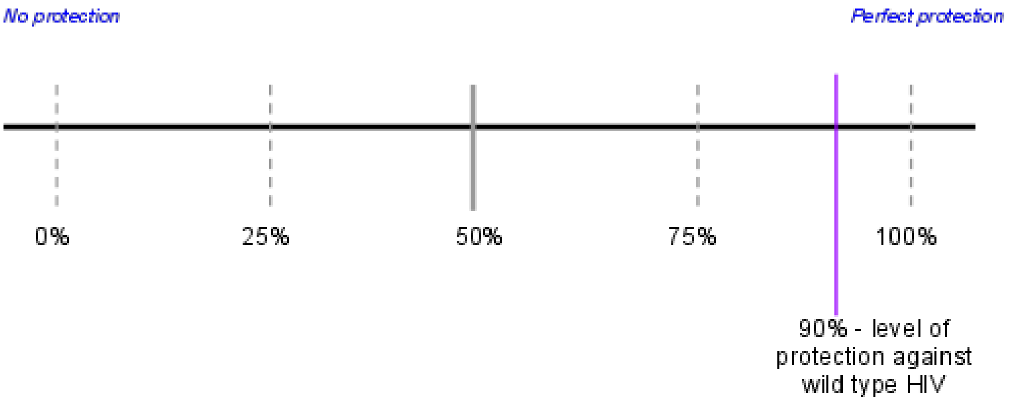

Comments:

3. Reversion of Drug Resistance

a) For an individual that had acquired drug-resistance whilst on PrEP but who now has not used PrEP what is the *proportion* of resistant virus compared to a total viral population over time after discontinue PrEP use.

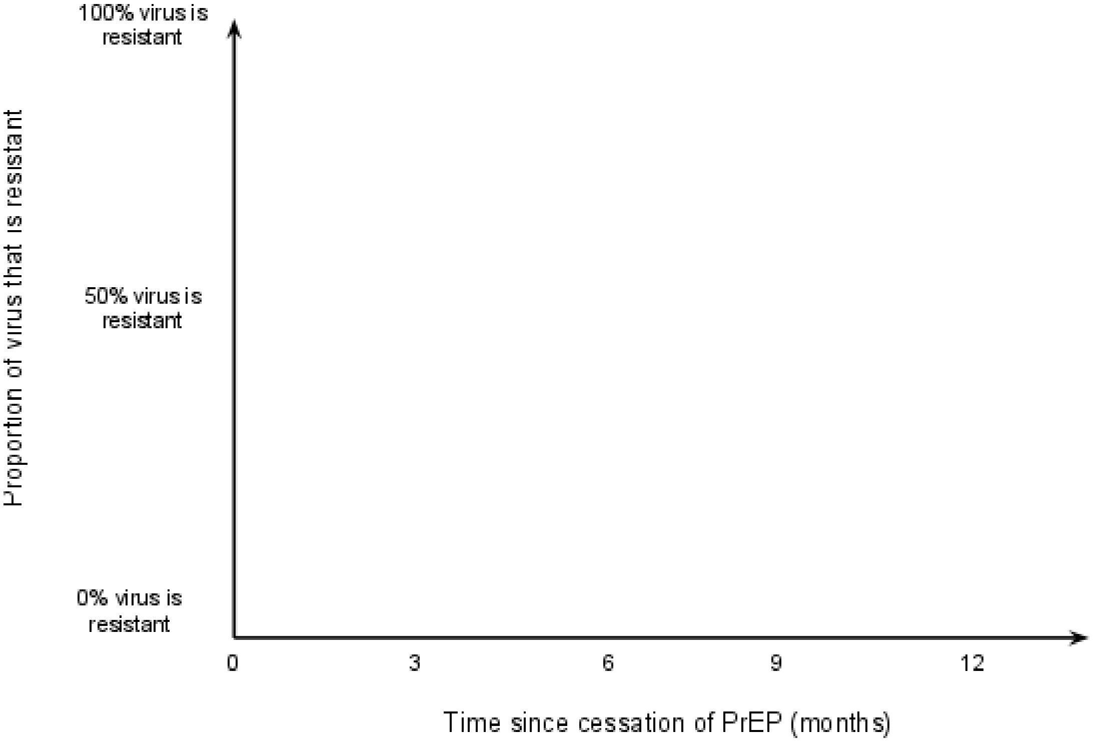

b) For an individual that has transmitted drug-resistance and never used PrEP what is the *proportion* of resistant virus compared to a total viral population over time after transmission. (Note: Insert graph)

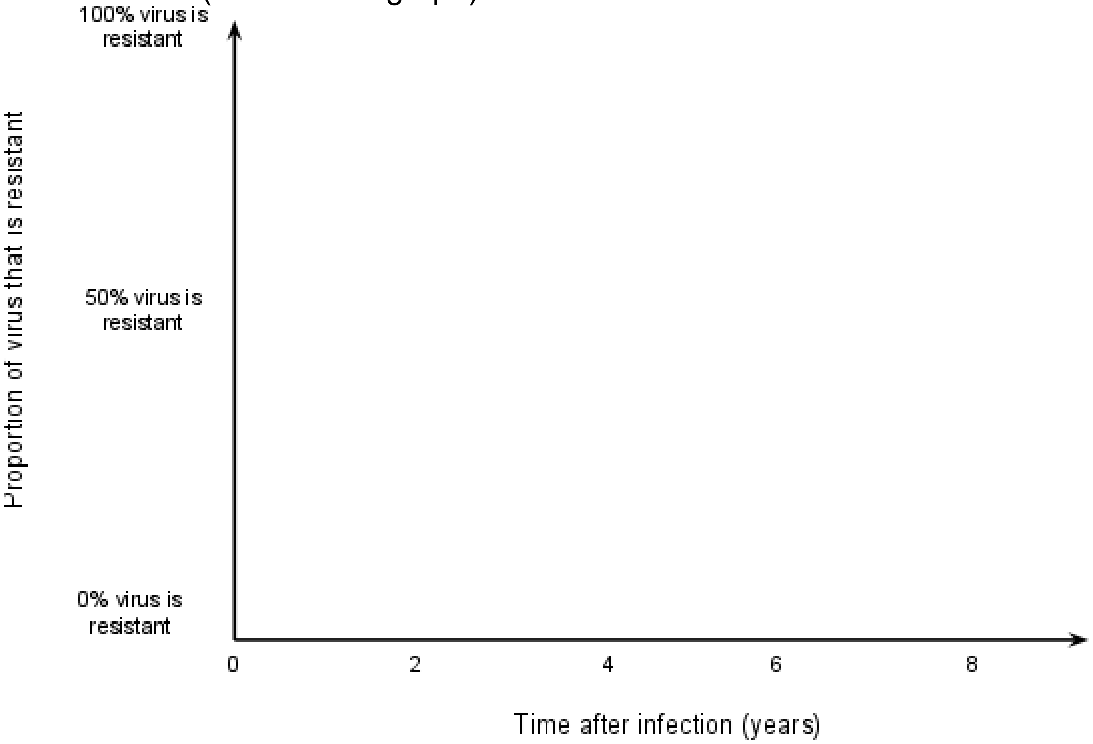

Comments:

4. Characteristic of Transmitted Drug Resistance

If drug-resistant HIV is transmitted, what will be relative chance of transmitting to someone else (compared to if the infection has been a wild-type virus: lower/same/higher), and what is the probability that the transmitted virus is resistant virus:

a) One week after infection;

Relative chance of transmission (of any type of virus) compared if infected with wild-type:

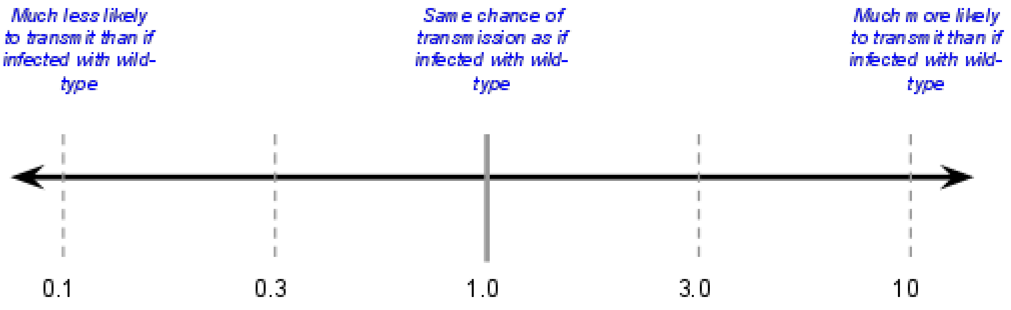

Probability that the transmitted virus is resistant virus (if transmission occurs):

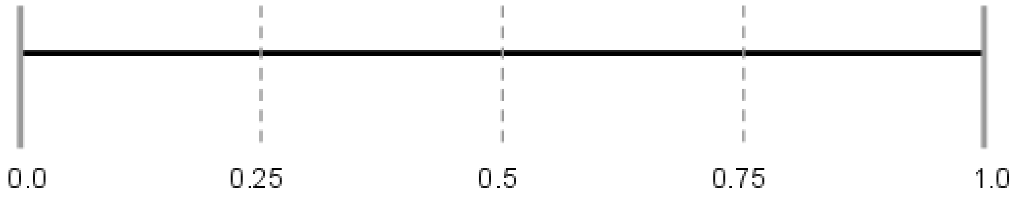

b) One year after infection;

Relative chance of transmission (of any type of virus) compared if infected with wild-type:

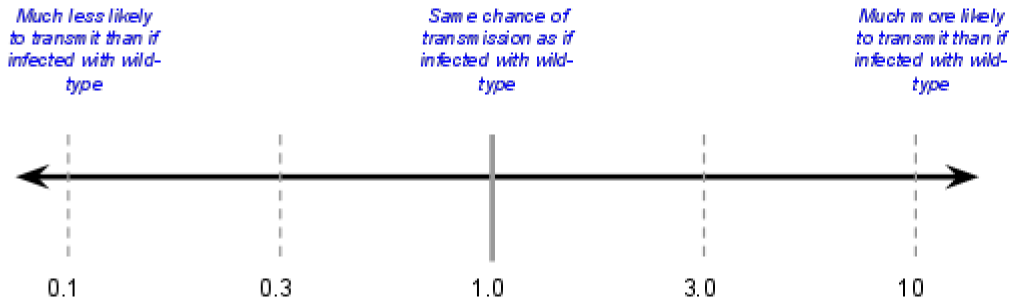

Probability that the transmitted virus is resistant virus (if transmission occurs):

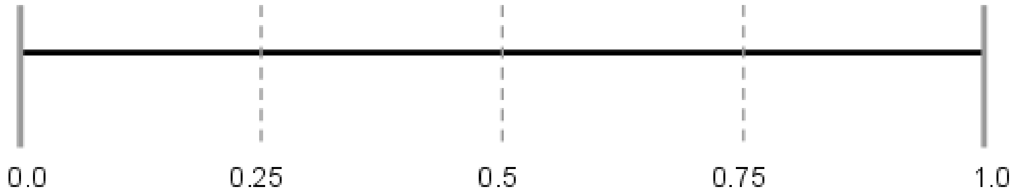

c) Five years after infection.

Relative chance of transmission (of any type of virus) compared if infected with wild-type:

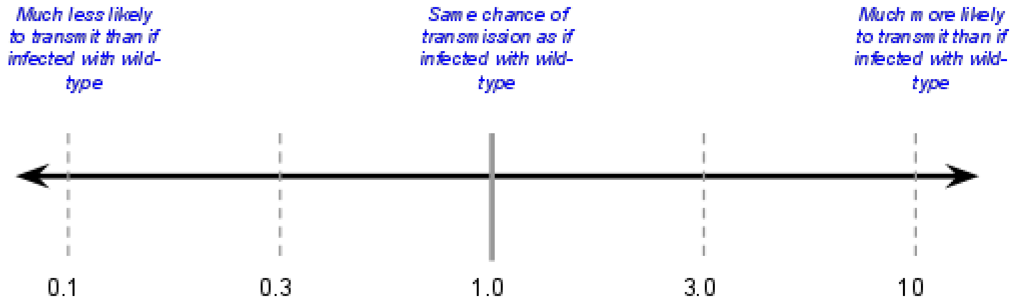

Probability that the transmitted virus is resistant virus (if transmission occurs):

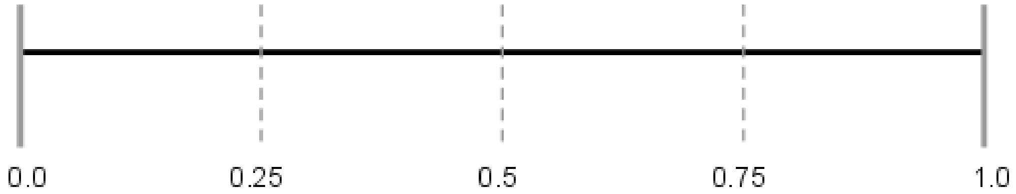

Comments:

5) Survival and disease progression when infected with drug-resistant HIV

a) In absence of ART what is the time from Infection to AIDS (shorter/same/longer cf wild-type) if infected with:

PrEP-Acquired drug-resistance:

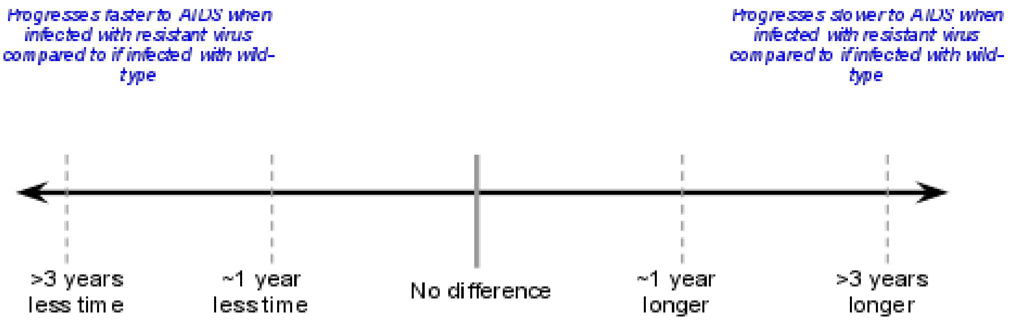

Transmitted drug-resistance:

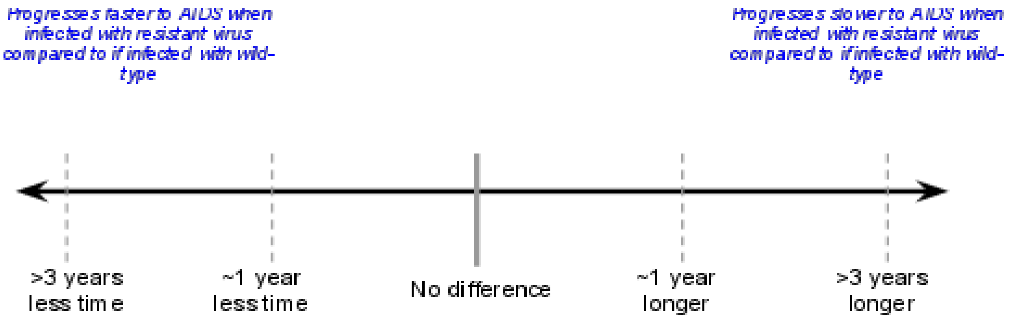

NB. The guidelines are not drawn to scale.

b) Probability of suppression on first-line ART in the first year. (lower/same/higher cf wild-type) if infected with:

Acquired drug-resistance:

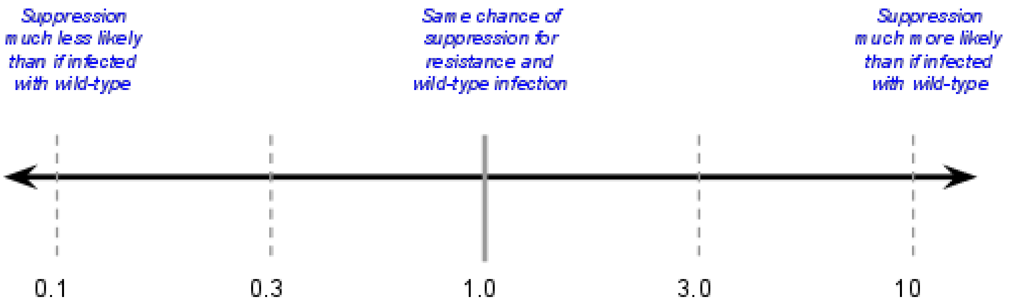

Transmitted drug-resistance:

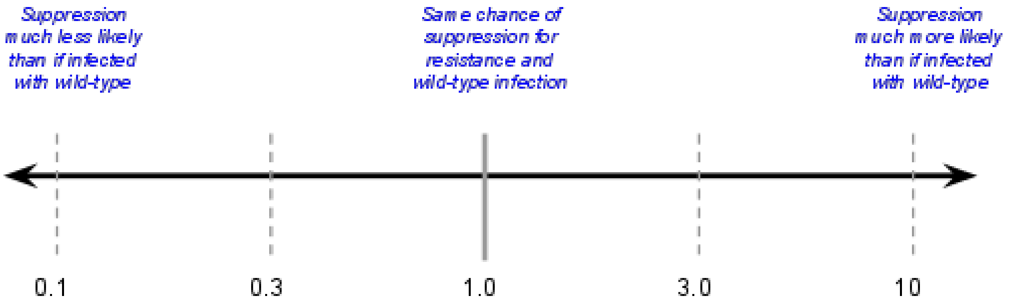

Comments:
